# Inflammation-fibrosis interplay in inflammatory bowel disease: mechanisms, progression, and therapeutic strategies

**DOI:** 10.3389/fphar.2025.1530797

**Published:** 2025-02-28

**Authors:** Yanan Li, Feng Xu, Yulai Fang, Yuan Cui, Zhenxing Zhu, Yuguang Wu, Yiheng Tong, Jingyi Hu, Lei Zhu, Hong Shen

**Affiliations:** ^1^ Department of Gastroenterology, Affiliated Hospital of Nanjing University of Chinese Medicine, Nanjing, China; ^2^ Department of Gastroenterology, The Third Affiliated Hospital of Zhejiang Chinese Medical University, Hangzhou, China; ^3^ Department of Gastroenterology, The Third Clinical Medical College of Zhejiang Chinese Medical University, Hangzhou, China; ^4^ Department of Gastroenterology, Ningxian second People's Hospital, Qing Yang, China

**Keywords:** intestinal fibrosis, inflammatory bowel disease, therapeutics, cellular mechanisms, molecular mechanisms, traditional Chinese medicine

## Abstract

**Background:**

The incidence of intestinal fibrosis in Inflammatory bowel disease has increased in recent years, and the repair process is complex, leading to substantial economic and social burdens. Therefore, understanding the pathogenesis of intestinal fibrosis and exploring potential therapeutic agents is crucial.

**Purpose:**

This article reviews the pathogenesis of IBD-related intestinal fibrosis, potential therapeutic targets, and the progress of research on Traditional Chinese Medicine (TCM) in inhibiting intestinal fibrosis. It also provides foundational data for developing innovative drugs to prevent intestinal fibrosis.

**Methods:**

This article reviews the literature from the past decade on advancements in the cellular and molecular mechanisms underlying intestinal fibrosis. Data for this systematic research were obtained from electronic databases including PubMed, CNKI, SciFinder, and Web of Science. Additionally, a comprehensive analysis was conducted on reports regarding the use of TCM for the treatment of intestinal fibrosis. The study synthesizes and summarizes the research findings, presenting key patterns and trends through relevant charts.

**Results:**

This study reviewed recent advancements in understanding the cellular and molecular mechanisms of intestinal fibrosis, the active ingredients of TCM that inhibit intestinal fibrosis, the efficacy of TCM formulae in preventing intestinal fibrosis, and dietary modification that may contribute to the inhibition of intestinal fibrosis.

**Conclusion:**

This article examines the cellular and molecular mechanisms that promote the development of intestinal fibrosis, as well as potential therapeutic targets for its treatment. It also provides a theoretical basis for exploring and utilizing TCM resources in the management of intestinal fibrosis. Through the analysis of various TCM medicines, this article underscores the clinical significance and therapeutic potential of TCM and dietary modifications in treating intestinal fibrosis.

## Graphical Abstract

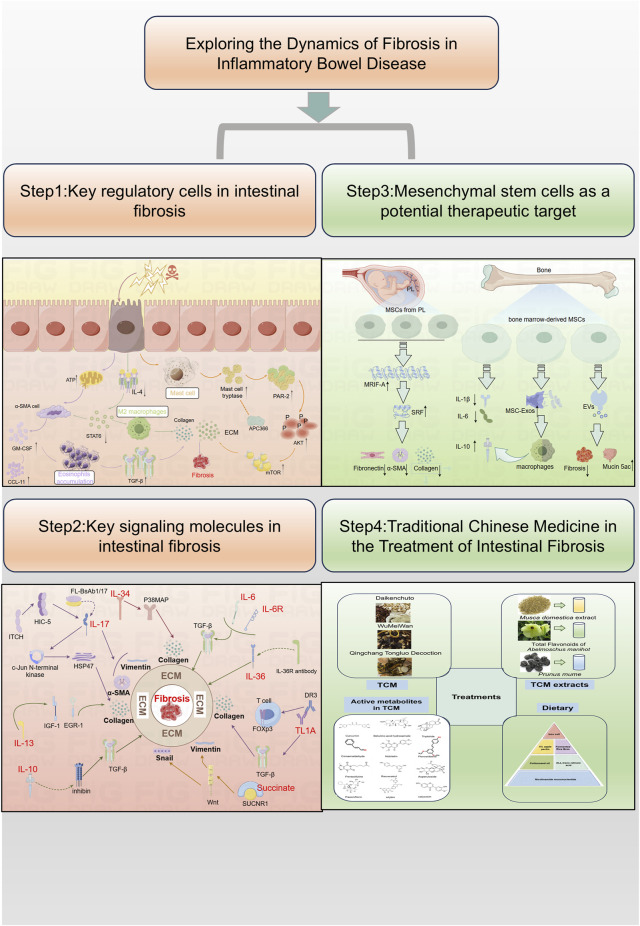

## 1 Introduction

Inflammatory bowel disease (IBD), which includes Crohn’s disease (CD) and ulcerative colitis (UC), is a chronic, relapsing inflammatory disorder (Baumgart and Carding, 2007; Ng et al., 2017). A severe complication of IBD is intestinal fibrosis, primarily characterized by the excessive accumulation of extracellular matrix (ECM) components (Rieder et al., 2017). The development of intestinal fibrosis is a pathological repair process in which persistent inflammation drives the sustained production of growth factors, proteolytic enzymes, and pro-fibrogenic cytokines (Rieder et al., 2024). These mediators activate ECM remodeling, leading to tissue damage and destruction (D'Haens et al., 2022). Ultimately, this process results in irreversible organ damage, loss of function, and bowel stenosis (Lenti and Di Sabatino, 2019).

Research has revealed that approximately 50% of CD patients develop strictures within 5 years of diagnosis, with more than one-third requiring surgical intervention (Spinelli et al., 2010). The incidence of strictures in CD increases significantly over time, rising from 7.2% at 1 year to 21.6% at 20 years (Lin et al., 2021; Liu et al., 2024). In contrast, the likelihood of stricture formation in UC ranges from 3.2% to 11.2%, with a 68% chance of occurring in the rectum (D'Alessio et al., 2022). Despite the growing burden of intestinal fibrosis, current therapeutic options, including biologics and endoscopic interventions, remain limited (Bettenworth et al., 2017; Bossuyt et al., 2022; de Buck van Overstraeten et al., 2012; Lan and Shen, 2018). Therefore, it is imperative to explore novel strategies and develop safe and effective therapeutic agents for the management of intestinal fibrosis.

Traditional Chinese Medicine (TCM) is known for its high efficacy, minimal side effects, and multi-target effects (Wang et al., 2021). This article provides an overview of the treatment of intestinal fibrosis with TCM, focusing on its active metabolites and their mechanisms of action. It further reviews the contributions of various cell types to the development of intestinal fibrosis, as well as the roles of cytokines and other molecular mediators in its pathogenesis. In addition, the article explores the potential for developing targeted therapeutic strategies based on TCM. Through an in-depth analysis of clinical studies on the use of TCM for the treatment of liver fibrosis and pulmonary fibrosis, this article aims to establish a theoretical foundation for the further development and application of TCM in advancing targeted therapies for intestinal fibrosis. By doing so, it provides new insights into potential treatment strategies for intestinal fibrosis.

## 2 Mechanisms that promote fibrosis in IBD

Following intestinal injury, an inflammatory response is triggered by the presence of bacteria, which can lead to increased fibroblast proliferation and the accumulation of ECM components, ultimately contributing to the exacerbation of scar formation. The detailed mechanisms by which inflammatory cells and cytokines promote intestinal fibrosis are summarized in Table 1.

**TABLE 1:** Literature on cellular and molecular mechanisms promoting intestinal fibrosis.

Category	name	Optimal doses	Source	Experimental model	Morphological aspects	Cytokine expression	Fibrotic changes	Mechanism of action	References
Inflammation cells	Eosinophil			RIF irradiation BALB/C mice			Azan staining↑; α-SMA↑; Collagen I↑; TGF-β↑	Eosinophil depletion suppresses small intestinal fibrosis	Takemura et al. (2018)
	Eosinophil		ASF	ASF -colonized C57BL/6 and BALB/C mice	Masson↑	IL-3↑; IL-11↓	VEGF↑; CXCL9↓	Microbial regulate enteric eosinophils increasing tissue remodeling	Jimenez-Saiz et al. (2020)
	MCs		Precursor cells→Mast cell	DSS induces C57BL/6 or Kit^Wsh^ mice; CCD-18Co fibroblasts; HMC-1 cells; LAD-2 cells	Colon length↑		MT staining↑; Collagen I↑; Tpsb2↑; Tpsb1↑; Fn1 ↑; α-SMA↑	MCs activate the protease receptor-2/Akt/mTOR pathway	Liu et al. (2021)
	MCs		MCT→ MCs	MCT or TGF-β1 induce CCD-18Co and LAD-2 cells	Masson↑		Vimentin↑; α-SMA↑; Fibronectin↑; Collagen I↑	MCs promote the differentiation of fibroblasts into fibrotic-phenotype myofibroblasts through Akt and Smad2/3 signaling pathways	Wan et al. (2022)
	M2 macrophage			TNBS induces SD Rat		CD163↑; D206↑; Caspase3↑; HIF-1α↑	Collagen I↑; Collagen III↑; PDGF-Rβ↓	M2 macrophages and intestinal smooth muscle cells are characterized by Inflammatory stricture formation	Lourenssen and Blennerhassett (2020)
	CD16^+^ macrophage	20 ng/mL	IL-4 activate STAT6	TNBS induces STAT6^−/−^mice	Masson↑	STAT3↑; FSP-1↑; CD16^+^↑	Collagen I↑; MMP2↑; TIMP-1↑; Vimentin↑; α-SMA↑; TGF-β↑	IL4-treated WT macrophages decrease CD16^+^ reducing fibrosis	Salvador et al. (2018)
Interleukins	IL-17	100 ng/mL	Th17cell→IL-17A	10 ng/mL TGF-β_1_ or IL-17A induces IEC-6 cell; TNBS induces fibrosis model with BALB/C mice	TJ↓; DAI↑; Colon shortening↑; Colonic injury↑	—	Vimentin↑; snail↑; FSP1↑; α-SMA↑; E-cadherin↓	—	Zhang et al. (2018)
	FL-BsAb1/17	10 mg/kg	IL-17/IL-1β antibody	DSS induces model with C57/BL6 mice	DAI↓; Colon shortening↓; Colonic injury↓; Masson↓	IL-1β↓; IL-6↓; TNF-α↓; IL-17A↓; IL-10↑; IL-12↓; IL-23↓; IL-22↓	TGF-β↓; α-SMA↓	—	Yin et al. (2021)
	IL-17	—	—	Itch^−/−^mice	Masson↑	—	α-SMA↑; Collagen I↑	Itch directly binds to HIC-5 and targets it for K63-linked ubiquitination to inhibit IL-17-driven intestinal fibrosis	Paul et al. (2018)
	IL-17	100 ng/mL		IL-17A induces CCD-18Co cell	—	—	HSP47↑; Collagen I↑	IL-17A via JNK pathway inductionHSP47 overexpression upregulated procollagen I and mature collagen I	Honzawa et al. (2014)
	IL-6	2 mg	IL-6-- IL-6R	Focal irradiation induces C57Bl/6J mice, and B6.129S2-Il6^tm1Kopf^/J mice	Colonic injury↑; Masson↑	CD4^+^↑; CD8^+^↓; Ki67↑; TNF-α↑	Picro Sirius Red↑	anti-IL-6R–treated mice presented with worsened intestinal fibrosis	Bell et al. (2019)
	IL-34	25–100 ng/mL	Recombinant human IL-34	IL-34 stimulated Intestinal fibroblasts	—	—	Collagen I↑; Collagen III↑	IL-34 stimulates gut fibroblasts to produce collagen synthesis	Franze et al. (2020)
	IL-10	5 μg/kg		Il-10^−/−^mice; WT 129/SvEv mice	Colonic injury↓; Masson↓	Nrf2↑ Prohibitin↑	α-SMA↓; Collagen I↓; TGF-β_1_↓	IL-10 treatment increased prohibitin expression, decrease fibrosis	Yuan et al. (2013)
	IL-36	250 μg/100 μL	IL-1→IL1RL2/L36R	DSS/TNBS induces model with IL1RL2^−/−^mice; L36R ligand inject Colonic lamina propria cells/Colonic fibroblasts cells	Colonic injury↓; Sirius Red↓	Ki67↑; IL36R↓	α-SMA↓; Collagen VI↓	IL1RL2^−/−^mice and mice given an injection of antibody against IL36R developed less fibrosis	Scheibe et al. (2019)
	IL-13 Inhibitor	100 μg	IL-13Rα_2_	TNBS induces model with BALB/C mice	Body weight↑; Masson↓		IGF-1↓; Collagen↓; EGR-1↓	IL-13 signaling via IL-13Rα_2_ induces fibrosis	Fichtner-Feigl et al. (2008)
TL1A	TL1A			L-Tg mice	HE↑	Foxp3↑; IL-2↓; IL-17A↓		Differential levels of Tl1a affect the expansion and function of regulatory T cells in modulating murine colitis	Sidhu-Varma et al. (2016)
	TL1Aantibody	500 µg		C57BL/6 mice; L-Tg mice; Transfer of CD4^+^CD45RBhigh T cell into RAG^−/−^mice	Masson↓		TGF-β1↓; Smad3↓	TL1A blocking ameliorates intestinal fibrosis in the T-cell transfer model of chronic colitis in mice	Li et al. (2018)
	TL1A			DSS induces WT or L-Tg mice; TL1A antibody/BMP-7 induced HT-29 cell	DAI↑; HE↑; Sirius Staining↑	IL-13↑	FSP1↑; α-SMA↑	Effect and Mechanism of TL1A Expression on Epithelial-Mesenchymal Transition during Chronic Colitis-Related Intestinal Fibrosis	Wenxiu et al. (2021)
	TL1A	100 ng/mL		TL1A induced MPFs; GF mice/GF-L-Tg mice	HE↑; Sirius Staining↑		α-SMA↑; Vimentin↑	Inflammation-independent TL1A-mediated Intestinal Fibrosis is Dependent on the Gut Microbiome	Jacob et al. (2018)
Succinate	Succinate	0.1–5 mM		TNBS induces WT and SUCNR1^−/−^ mice; 5 ng/mL TGF-β1 induces peritoneal macrophages		TNF-α↑; CD206↓	Collagen I↑; α-SMA↑; Vimentin↑	activates EMT in intestinal epithelial cells	Macias-Ceja et al. (2019b)
	Succinate	0.1–5 mM		Induction of Intestinal Fibrosis by Heterotopic Transplant of Colonic Tissue in WT and SUCNR1^−/−^ mice			Snail1↑; Snail2↑; E-cadherin↓; ITGB6↑; Vimentin↑	succinate and its receptor are upregulated around CD-fistulas and activate Wnt signaling and EMT in intestinal epithelial cells.	Ortiz-Masiá et al. (2020)
Other signaling molecules	deletion of Smad7			Heterotopic Intestinal Transplant Model; 3% DSS induced WT mice or Smad7^−/−^mice	Sirius↑; Masson↑	TNF-α↑; IL-6↑	Collagen V↑	Deletion of Smad7 Ameliorates Intestinal Inflammation and Contributes to Fibrosis	Schuler et al. (2023)
	ZNF281			10 ng/mL TGF-β induced CCD-18Co cell; DSS induced C57BL/6 male mice			α-SMA↑; FN1↑; Collagen I↑	ZNF281 Promotes Colon Fibroblast Activation in TGFβ1-Induced Gut Fibrosis	Laudadio et al. (2022)
	MAT			DNBS induced C57BL/6J mice; 3T3-L1 cell	HE↑; Colon damage↑	ATX↑; LPA↑; IL-6↑; IL-1β↑; TNF-α↑	TGF-β1↑; IGF1↑; VEGFA↑; CTGF↑	MAT contributes to intestinal fibrosis in Crohn’s disease through the ATX-LPA axis	Huang et al. (2022)
	microcystin-LR	1,60,120 μg/L; 60 μg/L	Environmental factors	C57BL/6 mice; NCM460 cell	HE↑; Masson↑	CSF1R↑; Rap1b↑; Nrf2↑; IL-6↑; IL-1β↑; TNF-α↑	TGF-β1↑; α-SMA↑; Collagen I↑	microcystin-LR exposure induces colorectal chronic inflammation, fibrosis and barrier disruption via CSF1R/Rap1b signaling pathway	Yang, Y. et al. (2022)

### 2.1 Key regulatory cells in intestinal fibrosis

**FIGURE 1:**
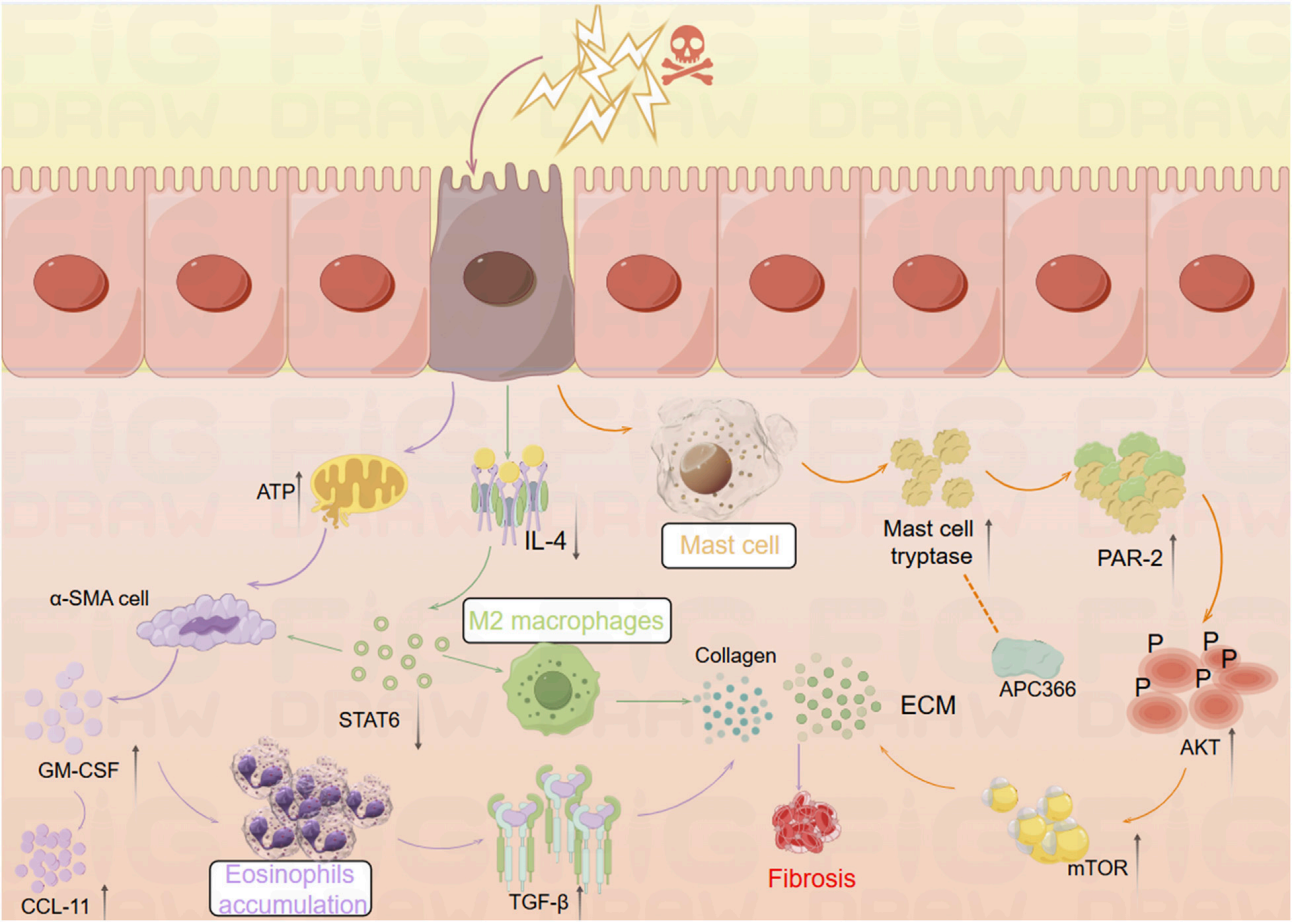
Mechanistic diagram illustrating the role of inflammatory cells in promoting intestinal fibrosis (By Figdraw). ① Intestinal inflammatory injury leads to an increase in α-SMA + cells, elevated GM-CSF levels, and increased eosinophils, which activate TGF-β1 and promote collagen deposition, contributing to fibrosis. ② Inflammatory stimuli increase mast cell tryptase levels, which, through the PAR-2/AKT/mTOR pathway, promote collagen deposition, a process that can be inhibited by APC366. ③ Loss of IL-4/STAT6 signaling results in an increase in M2 macrophages, which further promotes collagen fiber deposition. α-SMA, α-smooth muscle actin; ATP, adenosine triphosphate; APC366, Mast cell tryptase inhibitor; AKT, Protein Kinase B; CCL11, chemokine (C-C motif) ligand 11; ECM, extracellular matrix; GM-CSF, Granulocyte-Macrophage Colony-Stimulating Factor; IL-4, Interleukin-4; mTOR, mammalian target of rapamycin; PAR-2, Protease-Activated Receptor-2; STAT6, Signal Transducer and Activator of Transcription 6; TGF-β1, Transforming growth factor beta-1.

Inflammation is a non-specific response of the organism to injury, infection, or other stimulation, characterized by the activation, migration, and extravasation of inflammatory cells, along with local vascular changes (Medzhitov, 2010). Inflammatory cells play a central role in this process, with their extravasation serving as a critical hallmark of inflammation. These cells include leukocytes (mast cells), granulocytes (eosinophils), and monocytes (macrophages), among others (Abdulkhaleq et al., 2018). The pathway diagram in Figure 1 illustrates the involvement of inflammatory cells in the progression of intestinal fibrosis.

#### 2.1.1 Eosinophils

Eosinophils, derived from hematopoietic stem cells in the bone marrow, play diverse roles in maintaining tissue homeostasis (O'Sullivan and Bochner, 2018). Research has shown that Radiation-Induced Fibrosis (RIF) is mediated through the interaction between eosinophils and α-smooth muscle actin-positive (α-SMA) stromal cells (Larsen et al., 2007). Abdominal irradiation, particularly affecting the submucosal layer of the small intestine, leads to RIF and is associated with an excessive accumulation of eosinophils in both humans and mice (Wang et al., 2020). Following irradiation, chronic apoptosis of crypt cells elevates extracellular adenosine triphosphate (ATP) levels, which activates α-SMA cells within the submucosa. This activation induces the expression of Granulocyte-Macrophage Colony-Stimulating Factor (GM-CSF), which subsequently attracts eosinophils in mice. This interaction between eosinophils and α-SMA cells leads to the activation of chemokine (C-C motif) ligand 11 (CCL11) (Takemura et al., 2018). GM-CSF stimulates eosinophils to secrete Transforming Growth Factor-Beta 1 (TGF-β1), which promotes collagen expression in α-SMA cells. Inhibition of eosinophil accumulation post-irradiation through genetic deficiencies or neutralizing antibodies against C-C Chemokine Receptor Type 3 (CCR3) reduces the eosinophil influx in the submucosa of irradiated mice (Takemura et al., 2018). Additionally, studies have identified a close relationship between eosinophils and the gut microbiota. In germ-free (GF) mice, there is an increased presence of eosinophils in the small intestine, characterized by enhanced cytoplasmic protrusions and reduced granular content (Jimenez-Saiz et al., 2020). Eosinophil-deficient GF mice exhibit intestinal fibrosis and a reduced propensity for allergic reactions. These findings suggest that the commensal microbiota can modulate the frequency and function of intestinal eosinophils, thereby influencing tissue repair and fibrosis (Jimenez-Saiz et al., 2020).

#### 2.1.2 Mast cells

Mast cells (MCs) are key components of the sentinel immune cell population, playing a pivotal role in immune surveillance and tissue response (Abraham and St John, 2010). Recent studies have shown that IBD patients exhibit increased MC infiltration and enhanced expression of ECM proteins and genes (Liu et al., 2021). Furthermore, MCs have been implicated in the progression of inflammation and fibrosis in a TNBS-induced rat model of colitis (Xu et al., 2002). Mast cell tryptase (MCT), a serine protease stored in MC granules, is typically released in a degranulated form into fibrotic intestinal tissues, where it contributes to the enhanced expression of ECM proteins. The use of the tryptase inhibitor APC366 has been observed to reduce fibrosis progression (Liu et al., 2021). In co-culture experiments with CCD-18Co cells and MCs, treatment with tryptase was found to induce differentiation of fibroblasts into myofibroblasts and enhance ECM protein secretion. This process is primarily mediated through the activation of the Protease-Activated Receptor-2 (PAR-2)/Akt/mTOR signaling pathway (Liu et al., 2021). In a separate study, Wan et al. employed a decellularization process to remove native cellular remnants while preserving the natural components and structure of the ECM, creating natural decellularized intestinal scaffolds (DIS). The research demonstrated that transplanted fibroblasts could infiltrate these scaffolds and maintain their phenotype under both 2D and 3D culture conditions. Notably, MCT and TGF-β1 were found to promote the differentiation of fibroblasts into myofibroblasts via the Akt and Smad2/3 signaling pathways. In 3D culture conditions, the myofibroblasts produced significantly higher levels of Collagen type I and fibronectin synthesis compared to those in 2D culture (Wan et al., 2022). This study highlights that the potential of DIS as a biologically active microenvironment for studying intestinal fibrosis, providing a novel platform for simulating gut diseases and screening relevant genes and signaling pathways.

#### 2.1.3 Macrophages

A study has revealed significant phenotypic changes in M2 macrophages and intestinal α-SMA cells during the development of intestinal inflammatory strictures in rats (Lourenssen and Blennerhassett, 2020). Specifically, there was an increase in M2 macrophages expressing markers such as CD163, CD206, and arginase. Simultaneously, intestinal α-SMA cells proliferated continuously with low expression of platelet-derived growth factor receptor-beta (PDGF-Rβ). Over time, a selective and progressive increase in Collagen type I and III was observed (Lourenssen and Blennerhassett, 2020). Another study found that the deficiency of reparative M2 macrophages, which depend on the IL-4/STAT6 signaling pathway, led to delayed mucosal recovery in STAT6^−/−^ mice. This condition could be reversed through the exogenous transplantation of M2 macrophages (Salvador et al., 2018). Additionally, an increase in CD16^+^ macrophages was observed in the damaged mucosa of CD patients with stenotic or penetrating complications (Salvador et al., 2018). In bone marrow (BM) chimeric mice derived from CCR2-deficient mice, the study demonstrated the infiltration of CCR2^+^ BM-derived monocytes and fibroblasts into the colon, accompanied by an increased production of CCL2, which contributed to colonic fibrosis in BM chimeras. In contrast, chimeras with CCR2-deficient BM exhibited a reduction in fibrosis (Kuroda et al., 2019).

### 2.2 Key signaling molecules in intestinal fibrosis

**FIGURE 2:**
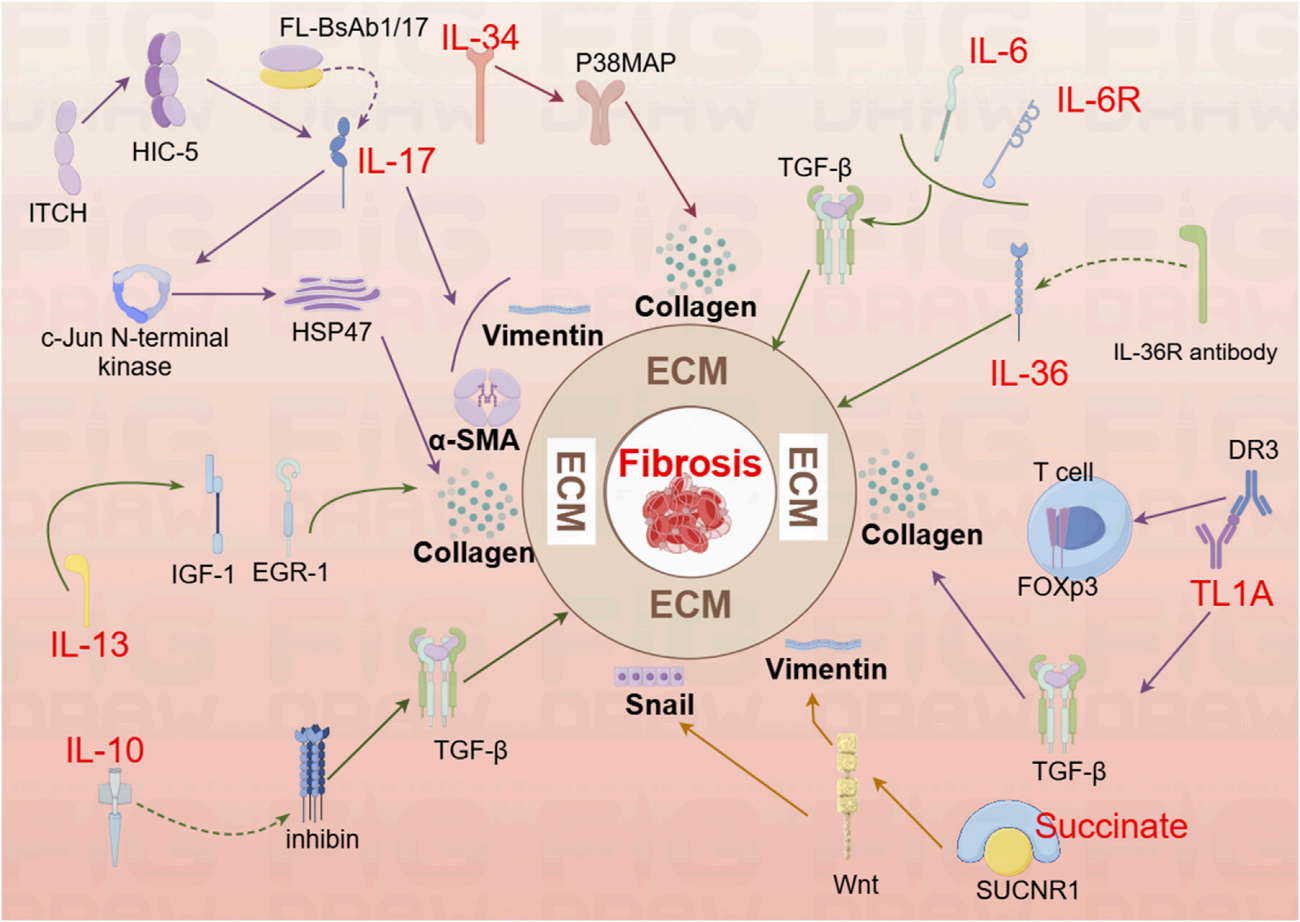
Mechanistic diagram illustrating the contribution of interleukin family cytokines, TL1A, and succinate to the development of intestinal fibrosis (By Figdraw). DR3, Death Receptor 3; EGR-1, early growth response gene −1; HIC-5, Hydrogen peroxide-inducible clone 5; HSP47, Heat shock protein; IL-6, Interleukin-6; IL-6R, Interleukin-6 Receptor; IL-10, interleukin 10; IL-13R, interleukin 13 Receptor; IL-36, interleukin 36; IL-36R, interleukin 36 Receptor antibody; IGF-1, colonic insulin-like growth factor-I; Itch, Itchy E3 ubiquitin-protein ligase; Snail, a transcription factor protein encoded by the snail gene; SUCNR1, succinate receptor; T cell, T lymphocyte; TL1A, Tumor Necrosis Factor-like Ligand 1A.

Cytokines are a diverse group of proteins that play a critical role in cell signaling and immune regulation (Wu et al., 2023). Inflammation is a fundamental pathological process by the tissue’s defensive response to various damaging stimulations (Schmitt et al., 2019). The chemical factors that mediate these inflammatory reactions are referred to as chemical or inflammatory mediators (Khan et al., 2023). Figure 2 provides a comprehensive overview of the mechanisms by which cytokines contribute to the progression of intestinal fibrosis.

#### 2.2.1 IL-17

Interleukin-17A (IL-17A), a key cytokine in the IL-17 family, is typically secreted as homodimers of IL-17A and IL-17F, or as heterodimers of the two (Schmitt et al., 2021). IL-17A was first identified in CD4^+^ T helper 17 (Th17) cells, with the differentiation of naive CD4^+^ T cells into Th17 cells following a three-step process (Beringer and Miossec, 2018). IL-17A plays a pivotal role in stimulating inflammatory responses in autoimmune diseases by promoting tissue inflammation, inducing the release of pro-inflammatory cytokines, and stimulating neutrophil chemotaxis. Additionally, IL-17A upregulates IL-6 in fibroblasts and preadipocytes, contributing to the pathogenesis of fibrosis (McGeachy et al., 2019). In a study by Zhang et al., analysis of intestinal mucosal biopsy tissues from CD patients revealed elevated IL-17A expression and increased occurrence of epithelial-mesenchymal transition (EMT). In IEC-6 cells, IL-17A was shown to induce ECM deposition, accompanied by a reduction in E-cadherin expression and an increase in Vimentin and α-SMA expression. *In vivo*, blocking IL-17A-mediated EMT induction alleviated intestinal fibrosis (Macias-Ceja et al., 2023; Zhang et al., 2018). Another animal study demonstrated elevated IL-17A levels in both the serum and intestines of mice, along with an upregulation of Collagen, TIMP-1, and MMP2. Treatment with anti-IL-17A antibodies resulted in reduced inflammation and fibrosis (Li et al., 2020). Further research showed that bi-specific antibodies targeting IL-1β and IL-17A (FL-BsAb1/17) administered to mice on alternate days led to downregulation of inflammatory cytokines, including IL-17A, IL-22, IL-1β, IL-23, in serum and colonic tissues (Yin et al., 2021). This treatment attenuated intestinal fibrosis, lowered transcription of Bax, and increased transcription of Bcl-2 in colonic tissues, thereby mitigating apoptosis (Yin et al., 2021). Moreover, the Itchy E3 ubiquitin-protein ligase (ITCH) has been identified as a key regulator in IL-17A-driven fibrosis. ITCH can ubiquitinate and target HIC-5 for degradation, thereby inhibiting the fibrogenic effects of IL-17A (Paul et al., 2018). HIC-5 plays a critical role by modulating myofibroblasts differentiation and ECM proteins expression (Lei et al., 2016). In mice, the ITCH gene deficiency leads to multi-organ inflammatory responses, and in humans, ITCH gene deficiency is associated with inflammatory diseases, including IBD (Venuprasad et al., 2015). Following IL-17A stimulation, myofibroblasts lacking ITCH exhibit increased expression of Collagen I and α-SMA. ITCH directly interacts with HIC-5, targeting it for K63-linked ubiquitination, which inhibits IL-17A-driven intestinal fibrosis (Paul et al., 2018). Additionally, IL-17A has been found to induce Heat Shock Protein 47 (HSP47), a key regulator of fibrosis (Honzawa et al., 2014). The expression levels of HSP47 and IL-17A were significantly elevated in the intestinal tissues of IBD patients during active disease phases, with a marked increase in HSP47-positive cells, particularly in α-SMA positive cells. IL-17A induces HSP47 protein expression in CCD-18 Co cells via the c-Jun N-terminal kinase (JNK) pathway (Honzawa et al., 2014). Thus, IL-17A represents a critical target in the development of intestinal fibrosis and warrants further investigation to uncover potential therapeutic mechanisms.

#### 2.2.2 IL-6

IL-6 is a pleiotropic cytokine with broad effects on various physiological processes, including the induction of inflammatory responses (Hunter and Jones, 2015). Inhibition of IL-6 signaling has been linked to improvements in fibrosis in both pulmonary (Le et al., 2014) and peritonea tissues (Fielding et al., 2014). However, a study investigating the effects of IR on the mouse small intestine revealed a dynamic immune cell infiltration pattern between days 3.5 and 14 post-IR, there was an increase in neutrophils, macrophages, B cells, and CD4^+^ T cells, while CD8^+^ T cells decreased between days 7 and 14. Notably, serum IL-6 levels significantly elevated following IR exposure. Surprisingly, IL-6 deficient mice (IL-6^−/−^) exhibited exacerbated intestinal damage after IR exposure, and mice treated with anti-IL-6R antibodies showed more severe fibrosis 2 months post-IR (Bell et al., 2019). These findings suggest a complex role for IL-6 in the context of intestinal injury, where its deficiency or inhibition may paradoxically aggravate tissue damage and fibrosis.

#### 2.2.3 IL-34

IL-34 is expressed in both the small intestine and colon in humans, with elevated levels observed in the inflamed intestinal tissues of patients with IBD (Liu Z.X. et al., 2022). Research has demonstrated that IL-34 promotes the migration and proliferation of synovial fibroblasts in patients with rheumatoid arthritis (Hwang et al., 2012) and stimulates the production of inflammatory cytokines in pulmonary fibroblasts (Zhou et al., 2018). A recent study further revealed increased RNA and protein expression of IL-34 and its receptor, M-CSFR-1, in the intestinal mucosa of patients with fibrostenotic CD (FS CD) compared to those with inflammatory CD (ICD). *In vitro*, IL-34 stimulation in normal fibroblasts resulted in upregulated expression of Collagen Iα1 and Collagen IIIα1, promoting collagen secretion and wound healing through a p-38 MAP kinase-dependent mechanism (Franze et al., 2020). These findings suggest that IL-34 plays a significant role in the fibrotic processes associated with CD and may serve as a potential therapeutic target for fibrotic complications in IBD.

#### 2.2.4 IL-10

The IL-10 cytokine family plays a pivotal role in the regulation of inflammation and disease progression (Ouyang et al., 2011). Recent studies have highlighted a depletion of mucosal IL-10 in CD patients, suggesting a potential mechanism for the disease’s chronic inflammatory nature (Carrasco et al., 2022). In an experimental model using IL-10 knockout mice for the study of intestinal fibrosis in CD, the expression of inhibin, a known suppressor of oxidative stress and lysosomal dysfunction with significant anti-inflammatory properties, was found to be involved. Specifically, in IL-10 knockout mice, an upregulation of inhibin expression was observed, which led to a reduction in the levels of inflammatory cytokines and TGF-β1. This modulation resulted in a significant alleviation of fibrosis, indicating that inhibin may play a crucial role in mitigating fibrosis in the context of IL-10 deficiency (Yuan et al., 2013). These findings suggest that IL-10 and its downstream signaling pathways, including inhibin, are critical in the regulation of fibrosis in CD and may represent potential therapeutic targets for managing intestinal fibrosis.

#### 2.2.5 IL-36

IL-36, a member of the IL-1 cytokine family known for its inflammatory effects, is expressed along with its receptor Interleukin-1 Receptor-Like 2 (IL1RL2 or IL36R), during the intestinal fibrosis processes in both humans and mice (Elias et al., 2021). Comparative studies have revealed that intestinal tissues from CD and UC patients exhibit higher levels of collagen, including collagen VI, compared to tissues from non-IBD patients. Notably, intestinal tissues from CD patients demonstrated an abundance of activated α-SMA, along with significantly elevated levels of IL-36A (Scheibe et al., 2019). Research has shown that the activation of IL-36R in fibroblasts from both mice and humans leads to the modulation of gene expression involved in fibrosis and tissue remodeling. Furthermore, IL1RL2 deficient mice (IL1RL2^−/−^), when treated with neutralizing antibodies against IL-36R, displayed reduced inflammation and alleviated intestinal fibrosis (Scheibe et al., 2019). A recent editorial mentioned that neutralizing antibodies targeting IL-36R are currently advancing to Phase II trials in patients with moderate to severe UC. In addition to their potential in treating chronic inflammation, these antibodies are also being explored as part of ongoing efforts to develop and validate anti-fibrotic therapeutic strategies (Mao and Rieder, 2019; Rieder et al., 2018). These findings underscore the emerging role of IL-36R as a promising target for both inflammatory and fibrotic disorders of the intestines.

#### 2.2.6 IL-13

The study revealed that IL-13 induces tissue fibrosis in colitis through a TGF-β1-dependent pathway (Fichtner-Feigl et al., 2007). Subsequent research demonstrated that IL-13 mediates its effects via signal transduction, achieved by inhibiting the promoter sequences of signal elements in IL-13 receptor α2 (IL-13Rα2) and the TGF-β1 receptor, using small interfering RNA (siRNA) or induced oligonucleotides. Notably, blocking IL-13 resulted in a suppression of colonic expression of Insulin-Like Growth Factor (IGF)-I and Early Growth Response Gene (EGR)-1, both of which are critical in initiating and sustaining fibrotic processes (Fichtner-Feigl et al., 2008). EGR-1 is particularly essential for the early stages of fibrotic (Bhattacharyya et al., 2013), while both EGR-1 and TGF-β1 play a key role in the later stages (Ke et al., 2016), driving collagen deposition by fibroblasts in the colon (Li Y. et al., 2021). These findings underscore the important role of IL-13 and its downstream signaling pathways, particularly through EGR-1 and TGF-β1, in the pathogenesis of intestinal fibrosis, highlighting potential therapeutic targets for fibrosis modulation in colitis.

#### 2.2.7 TL-1A

Tumor Necrosis Factor-like Ligand 1A (TL1A), a protein encoded by the Tumor Necrosis Factor Superfamily Member 15 (TNFSF15) gene, exhibits pro-inflammatory effects through T cells. Upon binding to its receptor, Death Receptor 3 (DR3), TL1A synergistically enhances T lymphocyte activation, promoting the polarization and effector functions of Th1 and Th17 cells (Aiba and Nakamura, 2013). Recent studies have identified TL1A as a species-specific susceptibility gene for IBD (Thiebaut et al., 2009), with increased expression observed in both human patients and mouse models of IBD (Sidhu-Varma et al., 2016). In LCK-CD2-TL1A-GFP transgenic mice (L-Tg), TL1A expression correlates with Foxp3 levels, showing lower expression in GFP-low T cells, and higher expression in GFP-high T cells (Sidhu-Varma et al., 2016). Chronic colitis models, established by transferring CD4^+^CD45RB^^^high T cells from wild-type (WT) or L-Tg mice into recombinase-activating gene-1 deficient (RAG^−/−^) mice, demonstrated that TL1A antibody treatment mitigated intestinal inflammation and fibrosis. This effect was linked to inhibition of intestinal fibroblast activation and reduced collagen synthesis through suppression of the TGF-β1/Smad3 signaling pathway (Li et al., 2018). Further studies on HT-29 cells revealed that TL1A antibody and BMP-7 could inhibit the epithelial-mesenchymal transition (EMT) induced by TL1A (Wenxiu et al., 2021). Additionally, TL1A has been observed to exacerbate spontaneous ileitis in the inflamed intestinal mucosa of IBD, as well as induced proximal colitis and fibrosis (Jacob et al., 2018). In this study, fecal material from healthy human donors and Specific Pathogen-Free (SPF) mice were used to colonize germ-free (GF) wild-type and TL1A-transgenic GF mice (TL1A-Tg). Results demonstrated that fecal material from SPF mice induced collagen deposition and fibroblast activation in TL1A-Tg mice, linking gut microbiota composition-specifically Ruminococcus and Salmonella-with increased fibrosis. Conversely, Spirochaetes showed an inverse relationship with fibrosis, highlighting the potential role of microbiota in modulating TL1A expression and intestinal fibrosis (Jacob et al., 2018). These findings underscore TL1A’s critical role in both the immune response and fibrosis development in IBD, suggesting it as a promising therapeutic target for intervention.

#### 2.2.8 Succinate

Succinate, an intermediate product of the tricarboxylic acid cycle, is also a metabolic byproduct of gut microbiota and accumulates in areas of inflammation. Its signaling, mediated through the succinate receptor (SUCNR1), plays a critical role in modulating immune functions (Gilissen et al., 2016). In CD patients, both serum and intestinal tissues exhibit elevated levels of succinate, alongside increased expression of the SUCNR1 (Macias-Ceja et al., 2019a). SUCNR1 mediates the expression of inflammatory cytokines in macrophages and co-localizes with CD86, CD206, and α-SMA^+^ cells in the human intestine. Notably, the absence of SUCNR1 has been shown to confer a protective effect against intestinal fibrosis (Macias-Ceja et al., 2019a). A 2020 study further investigated the expression levels of succinate and SUCNR1 in intestinal tissues of CD patients with stenotic (B2) and penetrating (B3) complications. Although increased expression of succinate and SUCNR1 was observed in patients with B3-CD, the primary focus is on transition cells within fistula walls and surrounding stromal cells. In WT mice post-transplantation, an upregulation of SUCNR1 and actin activation were noted, whereas these changes were absent in the SUCNR1 knockout (SUCNR1^−/−^) model. Additionally, activation of SUCNR1 in HT29 cells was found to induce Wnt signaling and the expression of genes associated with EMT (Ortiz-Masiá et al., 2020). These findings highlight the role of SUCNR1 in the pathogenesis of intestinal fibrosis, suggesting its potential as a therapeutic target in CD.

#### 2.2.9 Others signaling molecules

The TGF-β1/Smad signaling pathway is tightly regulated, with Smad7, a member of the Smad protein family, serving as a key negative regulator with this pathway. Smad7 exerts its effects in both the nucleus and cytoplasm through a variety of mechanisms (Hayashi et al., 1997). Research has revealed that deletion of Smad7 leads to an improvement in intestinal inflammation but also contributes to the progression of fibrosis (Schuler et al., 2023). This finding suggests that the inhibition of Smad7 could represent a novel therapeutic option for IBD. However, it is crucial to consider the potential for exacerbation of disease-associated fibrosis when targeting Smad7 therapeutically. Zinc-finger proteins (ZNFs) are among the most abundant protein families in the human genome (Cassandri et al., 2017), and ZNF281, a transcriptional regulator within this family, has been identified as a key factor in inducing EMT (Pieraccioli et al., 2016). Research has demonstrated that ZNF281 plays a role in promoting the activation of colon fibroblasts during TGFβ1-induced intestinal fibrosis (Laudadio et al., 2022). In mesenteric adipose tissue (MAT), the ATX-LPA axis has been shown to be activated in CD patients, with a positive correlation observed between its activation and both intestinal inflammation and fibrosis severity. Experimental validation demonstrated that the ATX inhibitor PF-8380 can mitigate intestinal fibrosis and inflammation. Furthermore, treatment of fibroblasts with 10 µM LPA significantly enhanced cell proliferation and differentiation into myofibroblasts (Huang et al., 2022). Another intriguing finding pertains to environmental factors: long-term exposure to microcystin-LR for 12 months, or after 40 cell passages, induces chronic inflammation, fibrosis, and barrier disruption in colorectal cells via the CSF1R/Rap1b signaling pathway (Yang Y. et al., 2022). These findings underscore the crucial role of cytokines in the promotion of intestinal fibrosis, and highlight the potential of cytokine antagonists as promising therapeutic agents for the treatment of intestinal fibrosis. Further drug development efforts are warranted to explore their clinical applications in IBD.

### 2.3 Key microbiota in intestinal fibrosis

The pathogenesis of intestinal fibrosis in IBD remains incompletely understood, but there is growing recognition of the significant role played by the microbiota and its metabolites (Bernardi et al., 2024). Studies have highlighted the involvement of the adherent-invasive *Escherichia coli* (AIEC) strain LF82, which can stably colonize the intestine and enhance the expression of the IL-33 receptor ST2 in intestinal epithelial cells through flagellin, thereby promoting significant fibrosis (Imai et al., 2019). Targeting AIEC and its downstream IL-33-ST2 signaling pathway presents a promising therapeutic strategy for managing intestinal fibrosis. Additionally, it has been confirmed that the AIEC strain LF82 induces collagen deposition and upregulates pro-fibrotic genes in human intestinal epithelial cells (IECs). This strain also amplifies TGF-β1-induced EMT and myofibroblast activation, exerting a pro-fibrotic effect in the intestine (Chokr et al., 2021). Furthermore, the gut microbiota has been shown to exacerbate colitis by promoting the ubiquitination of myeloid cells and the accumulation of the innate immune receptor STING (Larabi et al., 2020). Another study discovered that CD-associated AIEC suppresses the expression of let-7b in exosomes derived from IECs, which in turn modulates macrophage polarization, further exacerbating intestinal fibrosis (Xu et al., 2023).

In contrast, some microbial populations have been identified as having potential therapeutic effects in alleviating fibrosis. For example, a study demonstrated that invasive *Lactococcus lactis* (NCDO2118 FnBPA^+^) carrying the *Mycobacterium leprae* Hsp65 antigen (pXYCYT: Hsp65) mitigated TNBS-induced colitis and fibrosis (da Cunha et al., 2020). Furthermore, the soluble fraction of the multi-strain probiotic Vivomixx^®^ was shown to reduce fibrosis by downregulating the expression of collagen I and α-SMA via the TGF-β1/Smad signaling pathway (Lombardi et al., 2021). Notably, a 2021 study published in *Nature Medicine* reported that engineered yeast probiotics expressing the human P2Y2 purinergic receptor exhibited a 1000-fold increased sensitivity to extracellular adenosine triphosphate (eATP). This sensitivity, coupled with the secretion of the ATP-degrading enzyme apyrase, allowed the yeast to sense and respond to inflammation-inducing eATP, thereby suppressing intestinal inflammation, reducing fibrosis, and alleviating microbial dysbiosis in an IBD mouse model (Scott et al., 2021). These findings underscore the complex interplay between the microbiota and intestinal fibrosis, highlighting both detrimental and therapeutic microbial interactions that may be harnessed for future treatment strategies.

## 3 Therapeutic targets for intestinal fibrosis in IBD

Currently, there are no specific medications approved for the treatment of fibrosis in IBD. However, experimental validation targeting key signaling pathways in fibrosis offers promising prospects for the development of novel therapeutic strategies. Mesenchymal stem cells (MSCs) have emerged as a potential therapeutic approach due to their self-repair and regenerative properties, which can help prevent the onset and progression of intestinal fibrosis. Additionally, intracellular cytokines play a crucial role in maintaining the balance and stability of the ECM, thereby ameliorating the fibrotic process in the intestinal (Table 2). Parallel to research on intestinal fibrosis, therapeutic agents developed for fibrosis in other organs offer valuable insights for potential cross-application in IBD. For example, PPAR-γ agonists, such as Pioglitazone and Elafibranor, are currently undergoing Phase III clinical trials for the treatment of non-alcoholic steatohepatitis (NASH)-related liver fibrosis (Burns et al., 2021; Harrison et al., 2023; Schattenberg et al., 2021). Similarly, Pirfenidone, an established treatment for pulmonary fibrosis, has been extensively studied and approved by both the US FDA and the European Medicines Agency (EMA) for use in idiopathic pulmonary fibrosis (Behr et al., 2021; King et al., 2014; Noble et al., 2011). Given the common pathogenic mechanisms underlying fibrosis across different organs, we propose investigating the potential application of these drugs in the context of intestinal fibrosis. Such exploration could offer new therapeutic options and contribute to the development of more effective clinical strategies for the management of intestinal fibrosis in IBD.

**TABLE 2:** Therapeutic targets for intestinal fibrosis in IBD.

Category	Name	Optimal doses	Source	Experimental model	Morphological aspects	Cytokine expression		Fibrotic changes	Mechanism of action	References
MSCs	UC/PL-MSCs	1.0*10^5^ or 2.0*10^5^	UC/PL→MSCs	TGF-β induces HIMFs and UC/PL-MSCs				Collagen I↓; FN1↓; α-SMA↓; p-Smad2↓	Downregulated fibrogenesis by inhibition of RhoA, MRTF-A, and SRF expression	Choi et al. (2019)
	MSCs		The bone marrow cavity→MSCs	TNBS induces CD-associated fibrosis	Masson↓; Colon shortening↓; Colonic injury↓	IL-1β ↓; IL-6↓; IL-10↑; IL-13↓		E-cadherin↓; α-SMA↓; TGF-β↓; p-Smad2↓; p-Smad3↓	MSCs can treat fibrosis in CD	Lian et al. (2018)
	MSC-Exos		MSCs→ Exosomes	DSS induces IBD colitis and Exosomes were administrated to mice	DAI↓; Colon shortening↓; Colonic injury↓	IFN-γ↓; IL-1β↓; IL-6↓; TNF-α ↓; IL-10↑; CD206↑		—	Collagen catabolic process	Liu, H. et al. (2019)
	MSC-EVs		MSCs→ extracellular vesicles	Pro-inflammatory cytokine-induced MSCs/iMSCs; DSS- induced colitis	DAI↓; Colon shortening↓; Colonic injury↓; Sirius Red↓	IL-10↑; iNOS↓; MUC5ac↑		Collagen ↓	MSC-iEVs induced intestinal macrophages towards	Tolomeo et al. (2021)
	hADSCs	1.5*10^6^ cell	human adipose-derived MSCs	20 ng/mL IFN-γ and 50 µM Kynurenic acid induced hADSCs; TNBS induced Wistar Rats	HE↓; DAI↓; Colonic injury↓; Sirus Red↓; Masson↓	IL-1β↓; IL-6↓; TNF-α ↓; IL-10↑		Vimentin↓; α-SMA↓; laminin↓	Therapeutic efficacy of hADSCs in Crohn’s colon fbrosis is improved by IFN-γ and kynurenic acid priming through ido-1 signaling	Ye et al. (2022)
PPAR-γ	TRG RSG	TRG 25 μM; RSG 100 μM	PPAR-γ agonists→TRG/RSG	TGF-β_1_ induced HIFs				α-SMA↓; Collagen I↓; FN↓	PPAR-γ agonists decrease phosphorylation of Akt and Smad2 signally pathway	Koo et al. (2017)
	GED	30 mg/kg/d	PPAR-γ→ GED Agonist	2.5%DSS induced C57BL/6 mice; TGF-β induced IECs/HIFs/CCD-18Co cells	Colon length↑; Colonic injury↓; Masson↓	IL-13↓		Acta2↓; Collagen Ia1↓; FN1↓; α-SMA↓; CTGF↓	Ameliorates inflammation-driven intestinal fibrosis	Pompili et al. (2023), Speca et al. (2016)
Additional targets	Elafin	1 μg/mL	Human Endogenous Protein	SAMP1/YitFc mice; TNBS induce CD-1 mice; *Salmonella*. Induced 129 Sv/J mice; TGF-β induced CD-HIFs cell or CCD-18Co cell	Masson↓			Collagen I↓; Acta2↓; Vimentin↓	Elafin Reverses Intestinal Fibrosis by Inhibiting Cathepsin S-Mediated Protease-Activated Receptor 2	Xie et al. (2022)
	Adamdec1		ADAM metalloproteinase family	DSS induced WT mice or Adamdec1^−/−^ mice				α-SMA↓; FN1↓; Collagen I↓; Collagen VI↓	Colon stroma mediates an inflammation-driven fibroblastic response controlling matrix remodeling and healing	Jasso et al. (2022)
	AC-73	20 mg/kg	A small molecule that specifically disrupts CD147	TNBS induced BALB/C mice	HE↓; Sirius Staining↓	TNF-α↓; IL-6↓; IL-2↓; IL-17A↓; IL-12↓; IFN-γ↓; IL-34↓; IL-36α↓		Collagen I↓; Collagen III↓; CTGF↓; CD147↓	CD147 Targeting by AC-73 Induces Autophagy and Reduces Intestinal Fibrosis Associated with TNBS Chronic Colitis	Butera et al. (2022)
	NOX4			DSS induced NOX4^−/−^ mice		ROS↓; IL-1β↓; TNF-α↓		TGF-β1↓; Collagen Ia1↓; Collagen IIIa1↓	Role of Nox4 in Mitigating Inflammation and Fibrosis in DSS–Induced Colitis	Lee et al. (2023)
	MFGE8	3600 ng/µL 500 ng/mL		3.5% DSS induces mice; HIMFs	HE↓; Sirius Staining↓; Masson↓			Collagen I↓ FN↓; α-SMA↓	MFGE8 prevents intestinal fibrosis	Lin et al. (2024)
	Rapamycin	2 mg/kg	IL-23	TNBS induces model with C57BL/6 mice or Rag^−/−^mice	Colon change↑	IL-17↓; IL-22↓; IL-23↓; IL-1β↓		Collagen I↓; α-SMA↓; Collagen III↓; TGF-β↓	Rapamycin regulates the IL-23/IL-22 axis deceasing fibrotic	Mathur et al. (2019)
pirfenidone	pirfenidone	300 mg/kg 1 mg/mL		DSS induced C57BL/6J mice; TGF-β induced CCD-18Co cell		MAPK↓		Collagen I↓; Collagen III↓; TGF-β↓	Oral pirfenidone protects against fibrosis by inhibiting fibroblast proliferation	Li et al. (2016b)
	Pirfenidone	5 mg/kg 1 mg/mL		5ng/mL TGF-β induced InMyoFibs cell; TNBS induced TRPA1^−/−^mice	Masson↓	HSP47↓		α-SMA↓; Collagen Ia1↓; ACTA2↓	Activation of Myofibroblast TRPA1 by Steroids and Pirfenidone Ameliorates Fibrosis	Kurahara et al. (2018)
	Pirfenidone	100 mg/kg		Heterotopic Intestinal Transplant Model	Masson↓			MMP9↓; TGF-β↓	Decreased Fibrogenesis After Treatment with Pirfenidone	Meier et al. (2016)
	Pirfenidone	200 mg/kg or 400 mg/kg; 0.01–1mg/mL		Single-dose 20-Gy pelvic irradiation in SD Rats; TGF-β induces^58^RIFs				CTGF↓; Collagen I↓; α-SMA↓	Pirfenidone prevents radiation-induced intestinal fibrosis in rats	Sun, Y.W. et al. (2018b)
	Pirfenidone	1 mg/mL		10ng/mL TGF-β induced HIFs cell		p-13K↓; p-AKT↓		Collagen I↓; α-SMA↓	Pirfenidone suppresses TGF-β1-induced human intestinal fibroblasts	Sun, Y. et al. (2018)
	Pirfenidone	1 mg/mL		10ng/mL TGF-β induced HIFs cell		mTOR↓; p-70S6K↓		Collagen I↓; α-SMA↓; Collagen III↓; Collagen IV↓; Collagen VI↓; FN1↓	Pirfenidone Inhibits Cell Proliferation and Collagen I Production of Primary Human Intestinal Fibroblasts	Cui et al. (2020)
Angiotensin II	Losartan	7 mg/kg	Ang II	TNBS induces SD mice	Colon injury↓; Sirius Staining↓			TGF-β↓	Losartan reduces trinitrobenzene sulphonic acid-induced colorectal fibrosis in rats	Shi et al. (2016), Wengrower et al. (2012)
	Irbesartan	30 mg/kg		10-Gys RIF in C57BL/6J mice or CCR2^−/−^ mice	Sirius Staining↓	MCP-1↓; CCR2↓		Collagen I↓; MMP9↓	Irbesartan inhibits colitis-associated tumorigenesis by blocking the MCP-1/CCR2 pathway	Hachiya et al. (2021)

### 3.1 MSCs as a potential therapeutic target

**FIGURE 3:**
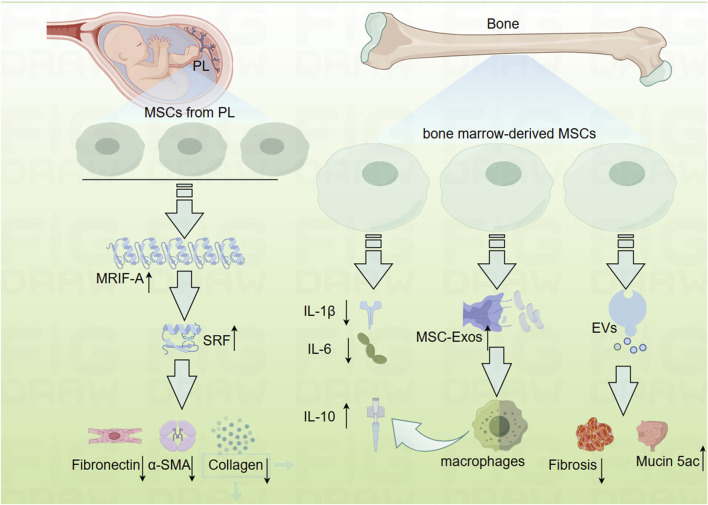
Mechanistic diagram depicting how mesenchymal cells inhibit the development of intestinal fibrosis (By Figdraw). EVs, mesenchymal stromal cells secreted extracellular vesicles; MRTF-A, Myocardin-Related Transcription Factor-A; MSC-Exos, human bone marrow-derived mesenchymal stromal cells; MSCs, mesenchymal cell; Mucin 5ac, oligomeric; PL, placenta; SRF, Serum Response Factor.

MSCs are multipotent stem cells characterized by their self-renewal capacity and potential for multilineage differentiation (Fu et al., 2019). Research has demonstrated that MSCs derived from perinatal tissues, including umbilical cords and placenta, can reduce fibrosis when applied to human primary intestinal myofibroblasts (HIMF) (Choi et al., 2019). The use of the small molecule inhibitor CCG-100602, which targets the Serum Response Factor (SRF) and its transcriptional coactivator Myocardin-Related Transcription Factor-A (MRTF-A), has further elucidated that the development of fibrosis is associated with the inhibition of the MRTF-A/SRF signaling pathway (Figure 3) (Choi et al., 2019).

In addition to perinatal MSCs, bone marrow-derived mesenchymal stem cells have also been explored for their potential in the treatment of IBD, although opinions regarding their efficacy remain divided. While several studies have highlighted the anti-inflammatory properties of bone marrow-derived MSCs (Goncalves Fda et al., 2014; Gonzalez et al., 2009; He et al., 2012), others have reported conflicting results (Duijvestein et al., 2011; Nam et al., 2015). Nevertheless, there is a broader consensus regarding the efficacy of MSCs in managing fibrosis (Figure 3). For instance, prophylactic administration of MSCs has been shown to inhibit fibrotic tissue accumulation, reduce fibronectin expression, and attenuate EMT. Additionally, therapeutic MSC application has demonstrated the ability to reverse established intestinal fibrosis and diminish EMT (Lian et al., 2018).

Further investigations have examined the role of MSC-derived exosomes (MSC-Exos) in treating IBD. One study revealed that systemic administration of human bone marrow-derived MSC exosomes targeting colonic macrophages significantly alleviated colitis in an IBD mouse model (Liu et al., 2019). Conversely, another study indicated that MSCs and their secreted extracellular vesicles (EVs) exert differential effects on experimental colitis. Bone marrow-derived MSCs were cultured under standard conditions (naïve, nMSCs) or preconditioned with IL-1β, IL-6, and TNF-α (induced, iMSCs), revealing that treatment with induced MSC-derived EVs resulted in reduced fibrosis, decreased angiogenesis, increased expression of Mucin 5ac, polarized intestinal macrophages, and a favorable shift in the Treg/Teff cells ratio in the intestinal lymph nodes (Tolomeo et al., 2021).

Moreover, human adipose-derived MSC (hADSCs), when preconditioned with IFN-γ and Kynurenic acid, have been shown to effectively ameliorate TNBS-induced colitis and colonic fibrosis. These hADSCs enhanced the indoleamine 2,3-dioxygenase 1 (IDO-1) signaling pathway, significantly inhibiting ECM deposition and EMT, while promoting the polarization of macrophages towards an anti-inflammatory M2 phenotype (Ye et al., 2022). These findings highlight the therapeutic potential of MSCs and their secreted products in modulating fibrosis and inflammation in IBD, warranting further exploration in clinical settings.

### 3.2 PPAR-γ agonists as a potential therapeutic target

Pioglitazone and Elafibranor, both Peroxisome Proliferator-Activated Receptor Gamma (PPAR-γ) agonists, have advanced to Phase III clinical trials for the treatment of Non-Alcoholic Steatohepatitis (NASH), underscoring the relevance of PPAR-γ in fibrotic processes across various organs (Zhang et al., 2009). PPAR-γ is recognized for its role as a natural protective agent against fibrosis, modulating both inflammatory and fibrotic pathways. Notably, a study demonstrated that co-incubation of TGF-β1 with PPAR-γ agonists, such as rosiglitazone or pioglitazone, resulted in a reduction in TGF-β1-induced expression of procollagen I, Fibronectin, and α-SMA. This downregulation of key fibrotic markers occurred through the inhibition of AKT and Smad2 phosphorylation, independent of the PPAR-γ pathway, highlighting a potential non-receptor-mediated mechanism of action (Koo et al., 2017). In addition to these established PPAR-γ agonists, a recent study has identified GED-0507-34, a novel PPAR-γ modulator, which has demonstrated antifibrotic effects in IEC cells induced by TGF-β1. GED-0507-34 was shown to significantly reduce the expression of TGF-β and ACTA1 in primary cultured intestinal myofibroblasts derived from UC patients, further supporting the therapeutic potential of PPAR-γ modulation in intestinal fibrosis (Speca et al., 2016). A comprehensive review of the anti-inflammatory and antifibrotic properties of PPAR-γ highlighted its multifaceted role in regulating the production of pro-inflammatory cytokines and interfering with profibrotic molecules such as Platelet-Derived Growth Factor (PDGF) and TGF-β (Pompili et al., 2023; Vetuschi et al., 2018). These findings underscore PPAR-γ as a critical target for the development of therapeutic strategies aimed at modulating both inflammatory and fibrotic pathways in diseases like IBD and NASH.

### 3.3 Additional targets for intestinal fibrosis in IBD

Elafin, a prototypical member of the WFDC (WAP [whey acidic protein] four-disulfide core) family of proteins, was initially characterized as a protease inhibitor, but further research has revealed its broader biological activities (Scott et al., 2011). Notably, studies have demonstrated that Elafin can reverse collagen synthesis in human intestinal tissues and myofibroblasts that have been preconditioned with exosomes derived from the serum of CD patients, highlighting its potential as an anti-fibrotic therapeutic (Xie et al., 2022).

Adamdec1, a member of the ADAM (A Disintegrin And Metalloproteinase) metalloproteinase family, has been shown to play a pivotal role in regulating ECM remodeling, intestinal healing, and inflammation (Lambrecht et al., 2018). Research has established that the deficiency of Adamdec1 accelerates ECM accumulation and disrupts tissue architecture, thereby contributing to impaired tissue repair and exacerbating fibrosis (Jasso et al., 2022).

The small molecule inhibitor AC-73 specifically targets CD147 dimerization, effectively blocking CD147’s action *in vivo* (Fu et al., 2016). CD147, also known as ECM metalloproteinase inducer (EMMPRIN), is a glycosylated transmembrane protein involved in ECM remodeling and regulation of wound healing, inflammatory diseases, and cancers (Landras et al., 2019). A study revealed that AC-73, by inhibiting CD147, effectively reduces intestinal fibrosis in a TNBS-induced chronic colitis model. This effect was mediated through suppression of the ERK1/2 and STAT3 signaling pathways, alongside induction of autophagy, highlighting CD147 as a potential therapeutic target for intestinal fibrosis (Butera et al., 2022).

Among the NADPH oxidase (NOX) family of enzymes, NOX4 is closely linked to tissue fibrosis, though its precise role in the fibrosis process remains controversial (Li Z.M. et al., 2021). Nevertheless, NOX4 has been implicated in modulating DSS-induced intestinal inflammation and fibrosis, with NOX4 deficiency exacerbating both inflammation and fibrosis, thereby hindering the recovery process (Lee et al., 2023).

Proteomic analysis of the decellularized gut has identified milk fat globule-epidermal growth factor 8(MFGE8) as a unique ECM protein in CD. MFGE8 was found to exert anti-fibrotic properties through integrin αvβ5 and focal adhesion kinase (FAK) in human intestinal myofibroblasts (HIMF), presenting a potential therapeutic pathway for mitigating fibrosis (Lin et al., 2024).

In a 2019 study, mTOR inhibitors, such as rapamycin, were shown to suppress IL-23 and IL-22 expression in TNBS-induced fibrotic mice, alleviating intestinal fibrosis. Further investigations revealed that deletion of the autophagy gene ATG7 increased IL-22 expression and exacerbated fibrosis. Additionally, inducing intestinal fibrosis with IL-22 in RAG^−/−^ mice demonstrated that depletion of innate lymphoid cells (ILCs) could mitigate fibrosis, suggesting that the pro-fibrotic process may occur independently of T and B cells (Mathur et al., 2019).

As clinical trials and experimental research on intestinal fibrosis continue to progress, an increasing array of therapeutic targets is emerging. These findings provide valuable insights into potential treatments for fibrosis in inflammatory bowel diseases, offering hope for more effective clinical management in the future.

### 3.4 Pirfenidone as potential agents for the treatment of intestinal fibrosis

Currently, there are no medications specifically targeting advanced fibrosis have been approved for clinical use (Mignini et al., 2024). However, Pirfenidone (5-methyl-N-phenyl-2-(1H)-pyridone, PFD) has emerged as a promising antifibrotic agent, demonstrating significant efficacy in both clinical trials and experimental animal studies (Duan et al., 2015; Rodriguez-Castellanos et al., 2015; Takeda et al., 2014). Preclinical investigations have shown that Pirfenidone, administered either orally or via enema to WT mice, effectively inhibits the activation of TGF-β1-related Smad and MAPK pathways both *in vivo* and *in vitro*. These findings suggest that Pirfenidone has a multifaceted mechanism of action in fibrotic processes (Li et al., 2016a; Li et al., 2016b).

Furthermore, studies have highlighted the role of the TRPA1 gene in fibrotic progression. Notably, both steroids (such as prednisolone) and Pirfenidone were found to reduce calcium influx in intestinal myofibroblast cell lines (InMyoFibs), leading to decreased expression of key fibrotic markers, such as HSP47, collagen I, and α-SMA. This effect was antagonized by the selective TRPA1 pathway blocker HC-030031, underscoring the involvement of TRPA1 in the fibrotic response (Kurahara et al., 2018). Increased expression of TRPA1 has been observed in the narrowed intestinal segments of CD patients, with cells positive for both TRPA1 and HSP47 concentrated in these constricted regions, both in human tissue and in TNBS-treated mice (Kurahara et al., 2018).

In additional experimental models, Pirfenidone has been shown to reduce fibrosis by inhibiting collagen deposition. In an ectopic small intestine model, treatment with Pirfenidone resulted in a reduction in collagen layer thickness and a decrease in the expression of TGF-β1 and MMP9, leading to a reduction in fibrosis (Meier et al., 2016). Moreover, Pirfenidone has been demonstrated to prevent radiation-induced intestinal fibrosis in rats by inhibiting fibroblast proliferation and differentiation, as well as suppressing the TGF-β1/Smad/CTGF signaling pathway (Sun Y.W. et al., 2018b). A similar study reported that Pirfenidone inhibits the proliferation and differentiation of primary small intestinal myofibroblasts (RIFs) in rats, through the suppression of the TGF-β1/Smad/CTGF signaling pathway, ultimately preventing fibrosis and reducing collagen deposition (Sun Y. W. et al., 2018a). Additionally, Pirfenidone has been shown to inhibit the proliferation and apoptosis of human intestinal fibroblasts (HIFs) induced by TGF-β1, via modulation of the Smad and PI3K/AKT signaling pathways (Sun Y. et al., 2018). Another study revealed that Pirfenidone suppresses HIF proliferation and collagen I production through the TGF-β1/Mtor/p70S6K signaling pathway (Cui et al., 2020). These findings collectively support the potential of Pirfenidone as an effective therapeutic agent for the treatment of intestinal fibrosis, providing insights into its mechanism of action and therapeutic promise in managing fibrosis-related conditions in IBD.

### 3.5 Angiotensin II receptor blockers as potential agents for the treatment of intestinal fibrosis

Angiotensin II (Ang II), the primary effector molecule of the renin-angiotensin system (RAS), plays a critical role in regulating blood pressure, electrolyte balance, and fluid homeostasis. In addition to its hemodynamic effects, Ang II functions as a potent pro-inflammatory mediator, promoting tissue inflammation through the upregulation of monocyte chemoattractant protein-1 (MCP-1) and other pro-inflammatory cytokines (Shi et al., 2016). Ang II exerts its biological effects primarily through binding Ang II type 1 receptor (AT1R), which is involved in various inflammatory and fibrotic processes. Angiotensin II receptor blockers (ARBs), such as Losartan and Irbesartan, have been investigated for their potential anti-fibrotic effects in inflammatory conditions. Losartan, in particular, has been shown to exert beneficial effects in the context of chronic colitis, demonstrating anti-fibrotic properties by reducing inflammation and modulating fibrotic pathways (Wengrower et al., 2012). More recent studies have highlighted the effects of Irbesartan, which has been shown to inhibit the production of MCP-1 and reduce the accumulation of Ly6C^+^CCR2^+^ monocytes and myofibroblasts in inflamed colonic tissue. Furthermore, Irbesartan downregulates the expression of key fibrotic markers, including collagen I and matrix metalloproteinase-9 (MMP9), thereby impeding the progression of intestinal fibrosis (Hachiya et al., 2021). These findings suggest that ARBs, particularly Irbesartan, may offer a promising therapeutic strategy for mitigating inflammation and fibrosis in conditions such as IBD.

## 4 TCM in the treatment of intestinal fibrosis

**FIGURE 4:**
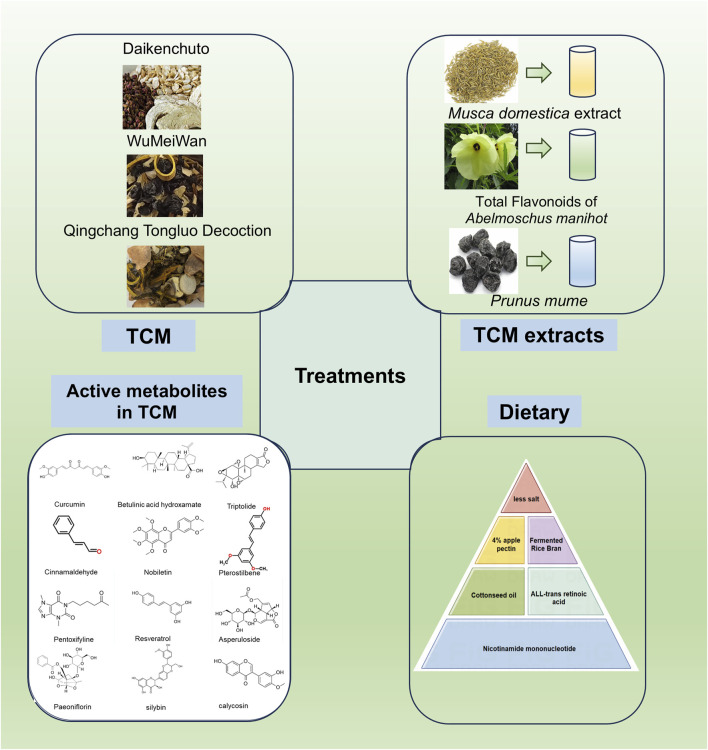
Effects of TCM, its extracts, and active metabolites of Chinese botanical drugs and dietary modulation on the improvement of intestinal fibrosis (By Figdraw).

TCM represents a profound and ancient system of medical knowledge that has significantly contributed to the promotion of health and the treatment of diseases for thousands of years. Notably, TCM has garnered increasing attention for its potential in managing complex conditions such as intestinal fibrosis. This therapeutic approach encompasses a wide range of strategies, including herbal formulations, extracts, and specific active metabolites derived from Chinese medicinal plants (Figure 4; Table 3, 4). TCM is distinguished by its simplicity, accessibility, cost-effectiveness, and efficiency, making it an attractive option for managing chronic and multifactorial diseases like intestinal fibrosis. The exploration of TCM-based botanical drugs and bioactive metabolites offers promising avenues for identifying novel therapeutic agents and strategies for the treatment of intestinal fibrosis. As such, further investigation into the mechanisms of action and clinical efficacy of these substances could provide valuable insights into new, integrative treatment options for this challenging condition.

**TABLE 3:** TCM formulations, its extracts, and active metabolites, as therapeutic targets for intestinal fibrosis.

Category	name	Optimal dose and administration schedule	Source	Experimental model	Morphological aspects	Cytokine expression	Fibrotic changes	Mechanism of action	References
TCM formulation	DKT	*In vivo*:5 mg/kg/d; 6 days *In vitro*:0.001%; 0.01%; 0.1%DKT; 24 h		TNBS induce TRPA1^−/−^ mice; 5 ng/mL TGF-β induced InMyoFib cell	Colonic injury↓; Masson↓		Collagen↓; Acta2↓	Activate myofibroblast TRPA1 to reduce fibrosis	Hiraishi et al. (2018), Inoue et al. (2011)
	Wu-Mei-Wan	0.48 g/mL; 0.96 g/mL; 1.92 g/mL 35 days		TNBS induces C57BL/6 mice	DAI↓; Masson↓		Collagen I↓; α-SMA↓; Fibronectin↓	Wu-Mei-Wan ameliorates chronic colitis-associated intestinal fibrosis through inhibiting fibroblast activation	Wu et al. (2020)
	QTF	*In vivo*:13.45 g/kg; 26.90 g/kg; 49 d *In vitro*:3.125 µg/mL, 6.25 µg/mL; 12.5 µg/mL; 24 h		TNBS induce BABL/C mice TGF-β induced IEC-6 cell	HE↓; Masson↓	IL-6↓; IL-1β↓; IL-17↓; IL-23↓	Collagen I↓; α-SMA↓; FN↓; TIMP1↓; VEGFA↓	Therapeutic Effects of Qingchang Tongluo Decoction on Intestinal Fibrosis in CD	Li et al. (2024)
TCM extracts	Maggot	*In vivo*:1 g/kg/d; 49 d *In vitro*:4µg/mL; 8 h	maggot→*Musca domestica* aqueous extraction	TGF-β1 induced CCD-18Co cells; DSS induced chronic colitis	Colonic injury↓; DAI↓	IL-6↓; IL-1β↓; TNF-α↓	Collagen I↓; α-SMA↓; Masson↓; TIMP1↓; pSmad2↓	Upregulate Nrf2 by suppressing TGF-β1/Smad pathway ameliorate fibrosis	Wang, R. et al. (2021)
	TFA	*In vivo*:250 mg/kg; 4w *In vitro*:5, 10 and 15 μg/mL; 48 h	TFA→*Abelmoschus Manihot;* ethanol extraction	TNBS induce BALB/C mice		TNF-α↓; IFN-γ↓; IL-6↓; IL-17↓; IL-10↑	TIMP-1↑; MMP9↓; Collagen I↓; Collagen III↓; α-SMA↓; Vimentin↓	TFA may restore the imbalance of Th17/Treg and decrease the generation of ECM	Qiao et al. (2021), Yang et al. (2018b)
	MFE40	135, 405 mg/kg; 10 d	MFE40→*Prunus mume*; 40% ethanol extraction	TNBS induces SD mice	HE↓; Masson↓	IL-6↓; IL-1β↓; IL-17↓	TGF-β1↓	MFE4 alleviates CD and its complications	Liu, Z. et al. (2022)
Active Metabolites in TCM	Curcumin	*In vitro*:2,5, 5, 10 μm; 7 d *In vivo*:50, 100, 200 mg/kg; 35 d	Curcumin → *Curcuma longa L*	TGF-β1 induced IEC-6 cells; TNBS induced rats	DAI↓; Colonic injury↓		α-SMA↓; E-Cadherin↓; p-Smad3↓; FN↓; CTGF↓	Curcumin Suppresses Intestinal Fibrosis by Inhibition of PPAR*γ*-Mediated EMT	Xu et al. (2017)
	silibinin	*In vivo*:100 mg/kg 200 days *In vitro*:5–100 μm 48–72 h	*Silybum marianum (L.) Gaertn*	IR induced C57BL/6 mice; TGF-β1 induced IEC-6 cells/CT26	Masson↓		α-SMA↓; TGF-β1↓; N-cadherin↓; E-cadherin↑	Suppress TGF-β1,p-MEK,p-ERK; p-p38 pathway	Kim et al. (2017)
	Calycosin	12.5–800 μmol/L; 24 h	Colycosin → *Astragalus mongholicus Bunge*	TGF-β1 induced CCD-18Co cells			Collagen I↓; α-SMA↓	regulate expression of TGF-β1/Smad signaling pathway	Liu, J. et al. (2019)
	BHA	*In vivo*:20、50 mg/kg; 14 days *In vitro*:5–20 μm; 24 h	Betulinic acid	TNBS model; DSS model; NIH-3T3-EPO-luc cell; Caco-2 cells	Colonic injury↓; Colon length↓	CD3^+^↓; F4/80^+^↓; IL-1β↓	Picrosirius red↓; TNC↓; TIMP1↓; MMP3↓; MMP8↓; Mrc-1↓	activate HIF-1 pathway	Prados et al. (2021)
	Triptolide	45mg/kg; 42 d	*Tripterygium wilfordii Hook.f*	TNBS induces SD mice	Masson↓		Collagen I↓; α-SMA↓; Vimentin↓	Triptolide ameliorates colonic fibrosis	Tao et al. (2015b)
	CCE or CCA	*In vivo*:4.5 mL/kg; 11w *In vitro*:0.1–10 µl/mL CCE; 5–500 µM CCA; 18 h	*Cinnamomum verum J.Presl;* ethanol extraction	WT and IL-10^−/−^ mice; 1μg/mL LPS induces HIFB cell or IMEF cell		IL-6↓; CXCL-2↓; CXCL-8↓	MMPs↓	Cinnamon reduces inflammatory response in intestinal fibroblasts	Hagenlocher et al. (2017)
	Nobiletin	*In vivo*:50 mg/kg; 11w *In vitro*: 5–100 µM; 24 h	*Citrus reticulata Blanco*	WT and IL-10^−/−^ mice; HIF cell	Masson↓; DAI↓; Colonic injury↓	IL-6↓; CXCL-2↓; TNF-α↓	Collagen I↓	Nobiletin acts anti-inflammatory on murine IL-10^−/−^ colitis and human intestinal fibroblasts	Hagenlocher et al. (2019)
	Pterostilbene	0.005%, 0.025%; 2w	*Vaccinium corymbosum L*	HFD and DSS induced C57bl/6J mice		IL-6↓; IL-1β↓	MMP2↓; TGF-β1↓	PTS is of significant interest for the prevention of HFD/DSS-induced colitis in C57BL/6J mice	Fan-Jiang et al. (2021)
	Pentoxifylline and its metabolite M-1	64 mg/kg; 10 d	*Theobroma cacao L*	TNBS induced mice	Masson↓; DAI↓; Colonic injury↓	MPO↓; IL-18↓	Collagen I↓	The effect of pentoxifylline and its metabolite-1 on inflammation and fibrosis	Peterson et al. (2011)
	Pentoxifylline and Vitamin-E	*In vitro*:100 µM–2 mM; 24 or 48 h *In vivo*:50 mg/kg; 20 d	*Theobroma cacao L*	5 ng/mL TGF-β1 induced HIMFs cell; DSS induced C57BL/6J mice		p-JNK↓; p-ERK↓	p-Smad2↓; Collagen Ia1↓; ACTA2↓; FN1↓; α-SMA↓	Combination of Pentoxifylline and Vitamin-E Inhibition of Intestinal Fibrosis	Lee (2022)
	Resveratrol	50 μM,100 μM 1-3 days	*Arachis hypogaea L*	1ng/mLTGF-β1 or 100ng/mL IGF-I induces smooth muscle cells isolated from colons of untreated Lewis rats			Collagen I↓; Collagen III↓	Resveratrol causes cell cycle arrest, decreased collagen synthesis, and apoptosis in rat intestinal smooth muscle cells	Garcia et al. (2012)
	Resveratrol	50 μmol/L; 100 μmol/L 24 h	*Arachis hypogaea L*	IGF-I induced CCD-18Co cells; TNBS induced SD mice		p-ERK1/2↓	Collagen I↓; IGF-I↓; SIRT1↓	Resveratrol inhibits collagenⅠsynthesis by suppressing IGF-1R activation in intestinal fibroblasts	Li et al. (2014)
	Asperuloside	40 µM; 24 h	*Galium odoratum (L.) Scop*	10 ng/mL TGF-β induces IEC-6 cell		TNF-α↓; IL-1β↓	Vimentin↓	Asperuloside inhibited epithelial-mesenchymal transition in colitis associated cancer via activation of vitamin D receptor	Lu et al. (2022)
	paeoniflorin	10,20,40 mg/kg; 10–12 w	*Paeonia lactiflora Pall*	TNBS and CUMS induced SD Rat	HE↓; Masson↓	p-P13K↓; p-AKT↓		Paeoniflorin Ameliorates Colonic Fibrosis in Rats with Postinfectious Irritable Bowel Syndrome by Inhibiting the Leptin/LepRb Pathway	Tian et al. (2022)

**TABLE 4:** The TCM formulas' specific components and therapeutic effects are outlined below.

Name	Components	Dosage forms and extraction method	Dosage	TCM effect	References
DKT (Jianzhong Decoction)	*Zingiber officinale Roscoe; Panax ginseng C.A.Mey. ; Zanthoxylum piperitum (L.) DC.*	Decoction; ethanol extraction	20 g	Warm the middle, tonify deficiency, and reduce counterflow to relieve pain	Hiraishi et al. (2018)
Wu-Mei-Wan	*Prunus mume (Siebold) Siebold & Zucc.; Asarum heterotropoides F.Schmidt; Zingiber officinale Roscoe; Coptis chinensis Franch.; Angelica sinensis (Oliv.) Diels; Typhonium trilobatum (L.) Schott; Zanthoxylum bungeanum Maxim.; Cinnamomum verum J.Presl; Panax ginseng C.A.Mey. ; Phellodendron chinense C.K.Schneid.*	Decoction; aqueous extraction	96 g	Strengthen spleen and kidney, clear away heat and resolve dampness, cool blood and resolve blood stasis	Wu et al. (2020)
QTF	*Scutellaria baicalensis Georgi; Paeonia officinalis subsp. Officinalis; Sophora flavescens Aiton; Phellodendron amurense Rupr.; Smilax glabra Roxb.; Angelica sinensis* var. *Sinensis; Angelica dahurica (Hoffm.) Benth. and Hook.f. Ex Franch. and Sav.;Citrus × aurantium L.;Atractylodes macrocephala Koidz.; Saposhnikovia divaricata (Turcz. Ex Ledeb.) Schischk.; Commiphora myrrha (T.Nees) Engl.; Vespa crabro; Vincetoxicum mukdenense Kitag.;Dioscorea oppositifolia L.; Glycyrrhiza uralensis Fisch. ex DC*	Decoction; aqueous extraction	182 g	Dispel dampness and detoxify, invigorate blood and resolve stasis, and nourish blood to unblock collaterals	Li et al. (2024)
Guizhi Jia Shaoyao Decoction	Cinnamomum verum J.Presl; Paeonia lactiflora Pall.; Glycyrrhiza uralensis Fisch. ex DC.; Ziziphus jujuba Mill.; Zingiber officinale Roscoe	Decoction; aqueous extraction	-	Warm the middle, tonify deficiency, harmonize the interior, and relieve stagnation	Author Anonymous (1987b)
Sanleng Wan	Sparganium stoloniferum (Buch.-Ham. ex Graebn.) Buch.-Ham. ex Juz.; Curcuma zedoaria (Christm.) Roscoe; Atractylodes lancea (Thunb.) DC.; Atractylodes macrocephala Koidz Coptis chinensis Franch	Decoction; aqueous extraction	43 g	Invigorate blood and eliminate stasis	Xu et al. (2017)
AHP	*Rehmannia glutinosa (Gaertn.) Libosch. ex DC.; Panax notoginseng (Burkill) F.H.Chen; Atractylodes macrocephala Koidz.; Paeonia × suffruticosa Andrews; Rheum officinale Baill*.*; Curcuma aromatica Salisb.; Hordeum vulgare L.; Acridocarpus plagiopterus Guill. and Perr.; Bombyx Batryticatus;Pheretima posthuman; Calculus bovis; Concha Arcae; Endothelium Corneum Gigeriae Galli; CornuBubali*	Granules	6 g	promoting blood circulation, resolving stasis, and tonifying the collateral system	Miao et al. (2019), Xiao et al. (2022)
Fuzheng Huayu Tablets	*miltiorrhiz Salvia a Bunge; Ophiocordyceps sinensis; Prunus persica (L.) Batsch; Gynostemma pentaphyllum (Thunb.) Makino;Schisandra chinensis (Turcz.) Baill*	Tablets	4.8 g	Invigorate blood and eliminate stasis, tonify essence and nourish the liver	Cheng et al. (2022)
Fufang Biejia Ruangan Tablets	Carapax trionycis; Curcuma longa L.; Paeonia lactiflora Pall.; Angelica sinensis (Oliv.) Diels; Panax notoginseng (Burkill) F.H.Chen; Codonopsis pilosula (Franch.) Nannf.; Astragalus mongholicus Bunge; Placenta hominis; Cordyceps sinensis; Saururus chinensis (Lour.) Baill.; Forsythia suspensa (Thunb.) Vahl	Tablets	6 g	Soften hardness to dissipate stagnation, and disperse blood stasis and detoxification, and enhance qi and blood	Rong et al. (2022)
YQSHD	*Astragalus mongholicus Bunge; Artemisia capillaris Thunb.;Scutellaria baicalensis Georgi;Coptis chinensis Franch.;Platycladus orientalis (L.) Franco;Curcuma aeruginosa Roxb.; Trionyxsinensis;Crataegus pinnatifida* var. *Pinnatifida;Paeonia lactiflora Pall.; Campsis grandiflora (Thunb.) K.Schum.;Atractylodes macrocephala Koidz.; Poria cocos(Schw.)Wolf.; Bupleurum chinense DC. ; Scleromitrion diffusum (Willd.) R.J.Wang*	Granules	5 g	Tonify Qi, invigorate blood, clear heat, detoxify, and eliminate dampness	Wu et al. (2021)
Mai Men Dong Decoction	*Ophiopogon japonicus (Thunb.) Ker Gawl.; Pinellia ternata (Thunb.) Makino; Panax ginseng C.A.Mey.* *Reynoutria japonica Houtt.; Angelica sinensis (Oliv.) Diels; Salvia miltiorrhiza Bunge; Glycyrrhiza glabra L*	Granules	70 g	Tonify Qi and Nourish Yin	Gan et al. (2020)
Qizhukangxian Granules	*Astragalus mongholicus Bunge;Curcuma phaeocaulis Valeton;Angelica sinensis (Oliv.) Diels* *Citrus hystrix DC.;Aster tataricus L.f.;Scutellaria baicalensis Georgi; Fritillaria monantha Migo; Glycyrrhiza uralensis Fisch. ex DC*	Granules	32 g	supplementing vital Qi combined with activating blood circulation, dispelling blood stasis and resolving phlegm	Guo et al. (2020)
Kangxianhuanji Granule	*Panax ginseng C.A.Mey.;Astragalus mongholicus Bunge;Gynostemma pentaphyllum (Thunb.) Makino* *Schisandra chinensis (Turcz.) Baill.; Epimedium brevicornu Maxim*	Granules	20 g	Tonifying Qi and Warming Yang	Hailong et al. (2022)
JHG	*Panax ginseng C.A.Mey.;Rehmannia glutinosa (Gaertn.) Libosch. ex DC.; Ophiopogon japonicus (Thunb.) Ker Gawl.; Trichosanthes kirilowii Maxim*.*; Fritillaria thunbergii Miq*.*; Paeonia × suffruticosa Andrews; Epimedium brevicornu Maxim*.*; Ginkgo biloba L*.*; Citrus reticulata Blanco;Glycyrrhiza uralensis Fisch. ex DC*	Granules	43.6 g	Tonifying Qi, Resolving Phlegm, and Invigorating Blood to Eliminate Stasis	Yang, S.G. et al. (2022)

### 4.1 TCM and its extracts

Recent studies have identified the unique efficacy of TCM and its bioactive metabolites in treating intestinal disorders, including intestinal fibrosis (Yang et al., 2021). One such example is Daikenchuto (DKT), a traditional Chinese herbal formula widely used in the management of gastrointestinal disorders (Katsuno et al., 2015). Research has demonstrated that oral administration of DKT alleviates intestinal fibrosis by reducing the expression of HSP47 protein and collagen content in the intestines (Inoue et al., 2011). Further investigation into the molecular mechanisms of DKT revealed its potential to modulate the Transient Receptor Potential Ankyrin 1 (TRPA1) pathway in intestinal myofibroblasts. TRPA1 knockout mice exhibited more severe inflammation and fibrosis, while DKT administration attenuated these fibrotic changes in WT mice, suggesting that DKT exerts its antifibrotic effects through activation of the TRPA1 pathway (Hiraishi et al., 2018). Calcium imaging assays revealed that DKT induced intracellular calcium influx in intestinal myofibroblasts (InMyoFibs), an effect that was reversed by the TRPA1 channel blocker HC-030031 (Hiraishi et al., 2018). Another notable TCM formulation, Wu-Mei-Wan, has demonstrated efficacy in ameliorating chronic colitis-associated intestinal fibrosis by inhibiting fibroblast activation (Wu et al., 2020). Additionally, Qingchang Tongluo Decoction (QTF), as validated by network pharmacology analysis, molecular docking, and both *in vivo* and *in vitro* experiments, has been shown to alleviate TNBS-induced intestinal fibrosis in mice by inhibiting the TGF-β/Smad/VEGF signaling pathway (Li et al., 2024).

Maggot extract (ME), derived from the larvae of *Musca domestica*, has been found to upregulate Nuclear Factor Erythroid 2–Related Factor 2 (Nrf2) and downregulate the TGF-β1/Smads pathway, thereby inhibiting intestinal fibrosis. The protective effects of ME were nullified upon inhibition of Nrf2, either by the Nrf2 inhibitor ML385 or siRNA targeting Nrf2, highlighting the role of Nrf2 in mediating its antifibrotic effects (Wang R. et al., 2021).

Total Flavonoids of *Abelmoschus manihot* (TFA), the main metabolite of the water extract of the traditional botanical drugs *A. manihot*, have shown significant therapeutic potential in conditions such as nephritis, nephrotic syndrome, and enteritis (Ge et al., 2016; Liu et al., 2017). TFA has been shown to enhance the expression of epithelial markers associated with fibrosis in IEC-6 cells through the TGF-β1 signaling pathway, reduce mesenchymal markers levels, and inactivate both the Smad and Mitogen-Activated Protein Kinase (MAPK) signaling pathways. The therapeutic efficacy of TFA was further augmented when combined with si-Smad and MAPK inhibitors (Yang et al., 2018a). Moreover, animal studies have confirmed that TFA improves intestinal fibrosis in mice by modulating the Th17/Treg cell balance and reducing ECM production (Qiao et al., 2021). These findings suggest that TFA may be a promising therapeutic agent for the treatment of intestinal fibrosis.

Citrate and hydroxycinnamate derivatives from *Prunus mume,* a common TCM remedy for chronic diarrhea and dysentery, have also shown promise in the management of CD and associated complications (Liu et al., 2023). A study demonstrated that the 40% ethanol fraction of *P. mume* (MFE40), the active fraction of its alcohol extract, effectively alleviates the inflammatory and fibrotic aspects of CD (Liu Z. et al., 2022). These findings underscore the potential of TCM and its bioactive metabolites as valuable therapeutic options for intestinal fibrosis, providing promising directions for future research and clinical application.

### 4.2 Active metabolites in TCM

Several studies have identified that specific monomeric metabolites possess notable potential in improving or alleviating fibrosis across various organs. Curcumin (1,7-bis(4-hydroxy-3-methoxyphenyl)-1,6-heptadiene-3,5-dione), a bioactive metabolite extracted from the rhizomes of the *Curcuma longa L.*, is well-known for its antioxidant, anti-inflammatory, antiviral, antiproliferative, and anticancer properties. Furthermore, Curcumin has shown promise in the prevention and treatment of autoimmune diseases, such as CD (Aggarwal and Harikumar, 2009). Previous research has highlighted the antifibrotic effects of curcumin in liver, lung, and cystic fibrosis (Lipecka et al., 2006; Nan et al., 2011; Punithavathi et al., 2000; Yao et al., 2012). Curcumin pre-treatment has been shown to significantly inhibit the expression of TGF-β and α-SMA genes in IEC-6 cells while promoting the expression of E-cadherin and PPAR-γ genes when compared to the positive control, rosiglitazone. Moreover, curcumin’s facilitation of PPAR-γ nuclear translocation can be reversed by the PPAR-γ antagonist GW9662, indicating its involvement in antifibrotic pathways (Xu et al., 2017).

Silybin, a natural polyphenolic flavonoid compound extracted from the fruits and seeds of *Silybum marianum (L.) Gaertn*., has been extensively applied in the treatment of colorectal cancer and antiproliferative therapies (Li et al., 2008; Ravichandran et al., 2010). Studies have demonstrated that Silybin ameliorates radiation-induced damage in mice by inhibiting the phosphorylation of Smad2/3, leading to a reduction in collagen deposition within the intestines and a decrease in TGF-β in both intestinal and plasma samples (Kim et al., 2017).

Calycosin, a key active metabolite of the traditional Chinese botanical drug *Astragalus mongholicus Bunge*, has exhibited multiple pharmacological activities and holds potential therapeutic efficacy against pulmonary and renal fibrosis (Chen et al., 2015; Deng et al., 2018; Duan et al., 2017). Compared to the positive control, 5-ASA, Calycosin effectively inhibits the expression of α-SMA and Collagen I in CCD-18Co by suppressing the TGF-β/Smad signaling pathway, further supporting its antifibrotic potential (Liu J. et al., 2019).

Betulinic acid hydroxamate (BHA), a pentacyclic triterpenoid compound, is an oxygen-mimetic derivative of betulinic acid, which has been shown to dose-dependently stimulate the activation of the Hypoxia-Inducible Factor (HIF) pathway in NIH-3T3 myofibroblasts, leading to collagen gel contraction (Prados et al., 2021).

Triptolide (PG490), a principal active metabolite of *Tripterygium wilfordii Hook.f.*, has been recognized for its antifibrotic effects in renal, hepatic, and pulmonary fibrosis (Chong et al., 2011; Krishna et al., 2001; Yuan et al., 2011). Research has demonstrated that treatment with Triptolide can reduce ECM deposition and collagen synthesis, and inhibit the expression of Collagen I, thereby alleviating fibrosis (Tao et al., 2015b).

Cinnamomum cassia extract (CCE), derived from the bark of the *Cinnamomum verum J.Presl*, contains Cinnamaldehyde (CCA) as its principal compound. CCA exhibits various beneficial biological effects, including the reduction of pro-inflammatory mediators in macrophages and monocytes (Chao et al., 2008). Moreover, both CCA and CCE have been shown to decrease the release of mast cell mediators, such as β-hexosaminidase and leukotriene C4, as well as pro-inflammatory factors like IL-6, KC/C-X-C motif ligand (CXCL) 8, and CCL2 in LPS-activated myofibroblasts, primarily by inhibiting the phosphorylation of I-kB (Hagenlocher et al., 2013; Hagenlocher et al., 2015). CCE treatment has been associated with a reduction in colonic fibrosis and collagen deposition in the colonic tissue of IL-10^−/−^ mice (Hagenlocher et al., 2017). In another study by the same research group, Nobiletin (3,4,5,6,7,8-hexamethoxyflavone), a polyethoxylated flavonoid compound found in the *Citrus reticulata Blanco*, was discovered to exhibit effects similar to CCA and CCE. It improves intestinal barrier function in a rat model of colitis and intervenes in the progression of fibrosis (Hagenlocher et al., 2019).

Pterostilbene, a natural stilbene compound in *Vaccinium corymbosum L.*, exhibits anticancer, anti-inflammatory, antifibrotic, and weight-reducing properties (Peng et al., 2022). Studies indicate that pterostilbene inhibits the expression of C/EBP homologous protein, Cyclooxygenase-2, and TGF-β1/Smad2/3, as well as MMP2, in the intestines of high-fat diet-fed (HFD) mice, suggesting its in modulating fibrosis (Fan-Jiang et al., 2021).

Pentoxifylline, a methylxanthine derivative, and its metabolite (1-(5-hydroxyhexyl)-3,7-dimethylxanthine, or metabolite-1, M-1), have been documented in the treatment of inflammation and fibrosis since the 1990s (Wen et al., 2017). Pentoxifylline and M-1 have been shown to reduce colonic inflammation and decrease collagen content, thereby mitigating fibrosis (Peterson et al., 2011). Moreover, combined treatment with Pentoxifylline and Vitamin E has demonstrated antifibrotic effects by inhibiting both Smad-dependent and Smad-independent TGF-β1 downstream signaling pathways, improving intestinal collagen fiber deposition in animal models. This improvement in intestinal collagen fiber deposition was also confirmed at the animal level (Lee, 2022).

Resveratrol (trans-3,5,4'-trihydroxystilbene), a phytoalexin found in berries, peanuts, grapes, and red wine, has been demonstrated to inhibit fibrosis in various organs, including the vasculature, heart, lungs, kidneys, and liver in animal models (Hong et al., 2010; Jing et al., 2010). In colon smooth muscle cells, resveratrol induced cell cycle arrest, increased the proportion of cells in the S phase, decreased collagen I expression, and inhibited TGF-β1-stimulated proliferation and IGF-I-stimulated collagen production, ameliorating intestinal fibrosis (Garcia et al., 2012). Additionally, in a colonic fibrosis model induced by TNBS in male SD rats, resveratrol reversed the increased expression of collagen I and IGF-I and decreased SIRT1 expression, with similar effects observed *in vitro* (Li et al., 2014).

Asperuloside, an iridoid compound from *Galium odoratum (L.) Scop*., is known for its anti-tumor, anti-inflammatory, antioxidant, and anti-obesity effects (Shen et al., 2023). Studies have demonstrated that Asperuloside inhibits EMT in colitis-associated cancer by activating the Vitamin D receptor/Smad3 pathway, thereby preventing the progression of EMT (Lu et al., 2022). Shaoyao (*Paeonia lactiflora Pall.)*, a traditional Chinese botanical drug with over 1,000 years of medicinal use, is commonly used for pain, inflammation, and immune disorders (Zhang and Wei, 2020). Paeoniflorin, an active metabolite from Shaoyao, has been shown to effectively ameliorate colonic fibrosis in rats with postinfectious irritable bowel syndrome by inhibiting the Leptin/Leptin Receptor b (LepRb) signaling pathway (Tian et al., 2022).

These findings underscore the potential of various monomeric metabolites derived from both traditional and modern sources as promising therapeutic agents for the treatment of intestinal fibrosis. Their multifaceted mechanisms of action highlight the importance of further investigation into these bioactive metabolites for the development of effective fibrotic disease therapies.

### 4.3 Acupuncture

Acupuncture, a cornerstone of TCM, has long been recognized for its therapeutic efficacy in managing various diseases (Zhuang et al., 2013). Recent studies have highlighted the potential of acupuncture-based treatments, such as mild moxibustion and herb-partitioned moxibustion (HPM), in addressing intestinal fibrosis in CD models. Specifically, it has been shown that these therapies can exert significant effects by modulating key signaling pathways involved in fibrotic processes. For example, mild moxibustion and HPM have been found to regulate the phosphorylation of Ras, Raf-1, MEK-1, and extracellular signal-regulated kinases (ERK-1/2) proteins in colonic tissues, contributing to the attenuation of fibrosis (Wang et al., 2013). Furthermore, HPM therapy has been demonstrated to reduce collagen deposition and improve fibrosis scoring in CD rats, likely through the modulation of the RhoA, ROCK1, and phosphorylated Myosin Light Chain (p-MLC) signaling pathways (Zhao et al., 2021). In addition, targeted moxibustion at specific acupoints, including Tianshu (ST25), Qihai (RN6), Zusanli (ST36), and Shangjuxu (ST32), has been shown to downregulate the expression of key fibrotic markers, including TGF-β1, Smad3, and Snail, while simultaneously inhibiting the expression of the mesenchymal marker fibronectin (Shi et al., 2019).These findings suggest that acupuncture, particularly HPM, may offer a valuable therapeutic approach for mitigating intestinal fibrosis, with potential mechanisms linked to the modulation of key molecular pathways involved in fibrosis development.

### 4.4 Clinical application of TCM in the treatment of fibrosis

Many TCM therapies, including individual botanical drug and herbal formulas, have been utilized throughout history to treat intestinal injuries and are believed to hold potential for restoring gut function. This underscores the need for further research to develop targeted anti-fibrotic therapies. In the 1980s, early case reports began to highlight the role of TCM in managing intestinal stenosis, which is considered the final clinical manifestation of intestinal fibrosis (Table 4). For instance, one case described a patient who, following multiple surgeries for intestinal obstruction, experienced recurrent adhesions leading to intestinal stenosis. The combination of DKT and Guizhi Jia Shaoyao Decoction was found to provide superior therapeutic outcomes in patients with cold deficiency syndrome (Author Anonymous, 1987b). Another case involved a female patient with a history of appendicitis, who underwent three surgeries in adulthood due to adhesions, intestinal stenosis, and perforation. After 3 years of treatment with Guizhi Jia Shaoyao Decoction, her symptoms were completely resolved, with no further discomfort (Author Anonymous, 1987a).

Recent clinical studies have continued to explore the efficacy of TCM in treating intestinal fibrosis. Notably, Xu Su and colleagues examined the effects of Sanleng Wan on intestinal fibrosis in patients with CD. Their findings indicated that patients treated with oral Sanleng Wan demonstrated significant improvements in endoscopic fibrosis scores, reduced CDAI scores, and lower levels of platelet count and D-dimer compared to those receiving conventional Biomedicine treatments like azathioprine (2 mg/kg/d) (Xu et al., 2017). Despite these promising results, progress in the use of TCM for intestinal fibrosis remains slower compared to advancements in the treatment of liver and pulmonary fibrosis. In contrast, significant research has been conducted on TCM’s role in treating liver and pulmonary fibrosis, where therapies have shown more rapid clinical application.

For liver fibrosis, TCM formulations with blood-activating and stasis-dispelling properties have been widely studied and applied, offering promising potential for preventing and reversing liver fibrosis. For example, the “Guidelines for Diagnosis and Treatment of Hepatic Fibrosis with Integrated Traditional Chinese and Biomedicine (2019 edition) (Xu et al., 2020), recommend several patented TCM formulations, including Anluo Huaxian Pills (AHP), Fuzheng Huayu Tablets (FZHY), and Fufang Biejia Ruangan Tablets (RGT). Randomized controlled trials have demonstrated the potential of RGT in reversing liver fibrosis in patients with chronic hepatitis B (CHB) or hepatitis B virus (HBV)-related compensated cirrhosis, although it does not prevent liver cancer (Qu et al., 2014). Furthermore, a multicenter trial published in 2022 highlighted the synergistic effects of RGT combined with entecavir (ETV), with patients showing an 8.2% higher rate of liver fibrosis regression after 72 weeks of treatment compared to controls (Rong et al., 2022). A multicenter trial with five hospitals showed that after 48 weeks, FZHY and ETV combination therapy did not differ significantly from the control in virological outcomes but improved biochemical responses and showed trends toward better liver fibrosis in hepatitis B-related cirrhosis patients (Cheng et al., 2022). A double-blind trial with 270 patients found significant histological improvement and reduced liver stiffness after 48 weeks of AHP treatment, with no severe side effects (Miao et al., 2019; Xiao et al., 2022). Another trial involving 802 hepatitis B-related cirrhosis patients found that YQSHD combined with entecavir reduced the annual liver cancer incidence to 1% over 2 years (Wu et al., 2021).

In the field of pulmonary fibrosis, numerous clinical trials have been conducted to explore the efficacy of TCM in managing conditions such as idiopathic pulmonary fibrosis (IPF). One randomized controlled trial involving 60 patients with IPF, characterized by Qi and Yin deficiency, demonstrated that treatment with Mai Men Dong Decoction combined with Pirfenidone for 24 weeks significantly improved pulmonary function and reduced the diffusion capacity for nitric oxide (DLCO) (Gan et al., 2020). Similarly, studies on Qizhukangxian Granules and Kangxianhuanji Granules have shown significant improvements in pulmonary function and symptom management in IPF patients (Guo et al., 2020; Hailong et al., 2022). A multicenter, double-blind trial with 312 IPF patients demonstrated that Jinshui Huanxian Granules (JHG) combined with Pirfenidone reduced acute exacerbations, improved pulmonary function, enhanced quality of life, and delayed disease progression after 52 weeks (Yang, S.G. et al., 2022). Another multicenter trial found that the combination of TCM mixed granules and Pirfenidone significantly improved respiratory function and quality of life in patients with post-COVID-19 pulmonary fibrosis (Lu et al., 2021).

These clinical studies underscore the importance of integrating TCM with synthetic drugs for the treatment of fibrosis across various organs. They also highlight the need for an evidence-based approach to evaluate the clinical efficacy of these integrative therapies. Moving forward, the development of a robust, multidimensional clinical efficacy evaluation system, which includes both TCM syndromes and patient quality of life, will be essential in advancing the treatment of fibrosis, particularly in the context of IBD. Ultimately, these studies may pave the way for a comprehensive treatment strategy that enhances the management of intestinal fibrosis and improves the quality of life for affected patients.

## 5 Modifying daily dietary habits to prevent intestinal fibrosis

Epidemiological studies have highlighted a strong correlation between the increasing incidence of IBD and changes in daily dietary habits, particularly the consumption of ultra-processed foods high in energy, salt, and additives, which can compromise the intestinal barrier. As living standards improve, dietary patterns have shifted towards more processed foods, contributing to gastrointestinal disturbances. Incorporating foods rich in vitamins, trace elements, and other beneficial nutrients, along with modifying dietary habits and supplementing with vitamin D, has been shown to positively influence the treatment of intestinal fibrosis (Table 5).

**TABLE 5:** Dietary, and vitamin D interventions as potential therapeutic targets for intestinal fibrosis.

Category	Name	Optimal doses(mg/kg,um)	Source	Experimental model	Morphological aspects	Cytokine expression	Fibrotic changes	Mechanism of action	References
Dietary	Salt	*In vivo*:4% w/w; *In vitro*:0-40 nM	Salt	TNBS induced SD mice; 10ng/mL TGF-β1 induced CCD-18Co cells			MMP2↑; TGF-β1↑; Collagen I↓; Collagen III↓; α-SMA↓	Dietary salt exacerbates intestinal fibrosis in chronic TNBS colitis via fibroblast activation	Amamou et al. (2021)
	Apple pectin	4% wt/wt	Soluble Dietary Fiber	Intestinal fibrosis induced by a single dose of abdominal irradiation of 10 Gy			α-SMA↓; Vimentin↓; Collagen I↓	Soluble Dietary Fiber Ameliorates Radiation-Induced Intestinal Epithelial-to-Mesenchymal Transition and Fibrosis	Yang et al. (2017)
	FRB	10%	rice	DSS induced mice	Masson↓; DAI↓; Colonic injury↓	IL-6↓; CXCL-2↓; TNF-α↓; IL-1β↓; INOS↓	MMP2↓; MMP3↓; Collagen I↓; Collagen I↓	Fermented Rice Bran Supplementation Prevents the Development of Intestinal Fibrosis	Agista et al. (2021)
	Cottonseed oil	200 μL	oil	DSS induced C57BL/6J mice	Masson↓; Colonic injury↓	IL-6↓; IL-17↓; TNF-α↓; IL-1β↓;^66^8-OHG↓; nltrotyosine↓	α-SMA↓; Collagen I↓	Cottonseed Oil Protects Against Intestinal Inflammation in DSS-induced IBD	Park et al. (2019)
	NMN	300 mg/kg/d	the precursor of NAD (+)	15Gy induced C57BL6/J mice	Masson↓		α-SMA↓; TGF-β ↓	NMN alleviates radiation-induced intestinal fibrosis by modulating gut microbiota	Zhao et al. (2023)
	FICZ, L-kynurenine, ITE, curcumin	FICZ:10–1,000 nM L-kynurenine:0.1–10 µM ITE:1–100 μM; curcumin:5–20 µM;	Dietary AhR Ligands	TGF-β1 induced CCD-18Co cells			No	Dietary AhR Ligands Have No Anti-Fibrotic Properties in TGF-β1-Stimulated Human Colonic Fibroblasts	Amamou et al. (2022)
Vitamin D	CARD-024	10–1000 nM	Vitamin D analog	1ng/mL TGF-β induced CCD-18Co cell		PTGS2↑; IL-1β↑	α-SMA↓; Collagen I↓; p-FAK↓; MLCK↓; ET-1↓	CARD-02 Attenuates the Pro-fibrotic Response to Substrate Stiffness in Colonic Myofibroblast	Johnson et al. (2012)
	Vitamin D	4.2IU/g	Vitamin D	TNBS induced CD-1 mice; SEMF cell	HE↓; Masson↓		TGF-β 1↓; p-Smad3↓; Collagen I↓	Vitamin D Prevents the Intestinal Fibrosis Via Induction of Vitamin D Receptor and Inhibition of Transforming Growth Factor-Beta1/Smad3 Pathway	Tao et al. (2015a)
	Vitamin D	*In vitro*:10 nM–100 nM *In vivo* 2 µg/kg; 2000U–4000 U		TGF-β induced CCD-18Co cell or HIFs; Heterotopic Intestinal Transplant Model; DSS induced VDR^−/−^ mice	Sirius Staining↓		α-SMA↓; Collagen Ia1↓; Vimentin↓; Fibronectin↓	Vitamin D receptor inhibits EMT via regulation of the epithelial mitochondrial function in intestinal fibrosis	Gisbert-Ferrándiz et al. (2020), Yu et al. (2021)

### 5.1 Dietary

Dietary habits play a significant role in the development and progression of intestinal fibrosis, with salt consumption being a critical factor. Excessive salt intake has been linked to various diseases, including hypertension and cardiovascular disorders (Kotchen et al., 2013). Studies have shown that high dietary salt exacerbates intestinal fibrosis in IBD, with experimental models indicating more severe intestinal inflammation and fibrosis in animals treated with TNBS and additional dietary salt (Amamou et al., 2021). In contrast, dietary interventions such as 4% apple pectin supplementation have been shown to reduce fibrosis markers by increasing short-chain fatty acids (SCFAs) and altering gut microbiota composition (Yang et al., 2017). Similarly, Fermented Rice Bran (FRB) and cottonseed oil have been demonstrated to modulate fibrosis-related pathways, reducing intestinal fibrosis in experimental models (Agista et al., 2021; Park et al., 2019). Additionally, compounds like All-trans retinoic acid (ATRA) and nicotinamide mononucleotide (NMN) have been shown potential to mitigate fibrosis in radiation-induced models, highlighting the role of dietary factors in the modulation of intestinal fibrosis (Leem et al., 2017; Okoshi et al., 2008; Zhao et al., 2023). Furthermore, Aryl Hydrocarbon Receptor (AHR) ligands, such as FICZ, curcumin, and L-kynurenine, have been explored for their anti-fibrotic properties, although some studies report contrasting findings regarding their effectiveness in human colonic fibroblasts (Amamou et al., 2022; Lamas et al., 2018; Lamas et al., 2016).

### 5.2 Vitamin D

The vitamin D analog CARD-024 (1-alpha-hydroxyvitamin D5) has been characterized as a compound with minimal hypercalcemic effects, making it a promising candidate for therapeutic use in fibrotic disease (Mehta et al., 2003). *In vitro* studies have demonstrated that CARD-024 can reverse the fibrotic phenotype induced by TGF-β stimulation in CCD-18Co colonic myofibroblasts, characterized by increased actin stress fibers, maturation, and elevated α-SMA expression, thereby ameliorating intestinal fibrosis (Johnson et al., 2012). Additional research has highlighted that vitamin D supplementation in vitamin D-deficient CD-1 mice resulted in downregulation of ECM production and collagen synthesis, as well as reduced TGF-β1 and Smad3 levels in subepithelial myofibroblasts isolated from mouse colon (SEMF) (Tao et al., 2015a). Furthermore, a reduction in vitamin D Receptor (VDR) levels has been observed in resected tissues and epithelial cells of CD patients, with vitamin D treatment found to restore VDR expression, preventing the accelerated transition of myofibroblasts and inhibiting intestinal fibrosis in an animal model (Gisbert-Ferrándiz et al., 2020). Notably, VDR knockout mice developed more severe intestinal fibrosis, and reduced VDR expression in human colonic myofibroblasts, suggesting that VDR plays a crucial role in the modulation of intestinal fibrosis (Yu et al., 2021). These findings suggest that vitamin D supplementation may serve as a potential strategy for preventing intestinal fibrosis.

## 6 Discussion

Intestinal fibrosis, a frequent complication of IBD, arises as a consequence of chronic inflammation and presents significant therapeutic challenges. This review has explored both cellular and molecular mechanisms underlying the promotion of intestinal fibrosis, as well as potential therapeutic targets. At the cellular level, processes such as epithelial-to-mesenchymal transition (EMT), endothelial-to-mesenchymal transition (EndMT), and the involvement of myofibroblasts have been well-documented; however, our focus has extended to include the contribution of immune cells such as eosinophils, macrophages, and mast cells in the fibrotic process (Figure 1). On the molecular front, in addition to inflammatory factors and TL1A, alterations in the gut microbiota and metabolites like succinate have emerged as key players in the progression of intestinal fibrosis (Figure 2). Notably, fibrosis can persist even in the absence of active inflammation, a phenomenon increasingly recognized in IBD. The coexistence of inflammation and fibrosis in IBD remains a challenge, as current diagnostic technologies, including CT, MRE, and intestinal ultrasound, often fail to clearly distinguish between these two changes. Therefore, the development of diagnostic techniques capable of accurately differentiating between inflammatory and fibrotic changes is essential for tailoring effective treatments.

From a therapeutic standpoint, the role of mesenchymal stem cells (MSCs) in modulating fibrosis has garnered considerable attention (Figure 3). While clinical randomized controlled trials are still lacking, preclinical studies have shown promising results. Furthermore, several molecular targets critical for intestinal fibrosis have been identified, and inhibitors or activators of these pathways have demonstrated significant effects in attenuating fibrosis. While research on intestinal fibrosis is still emerging, lessons learned from the more advanced studies on liver and pulmonary fibrosis provide valuable insights. For instance, clinically approved PPAR-γ agonists for liver fibrosis and therapies like Pirfenidone for pulmonary fibrosis offer potential strategies for treating intestinal fibrosis. Although preclinical studies suggest the applicability of these treatments to intestinal fibrosis, clinical validation is still required.

TCM offers an alternative approach to fibrosis treatment with a long history of use in China. TCM formulas, extracts, and metabolites are increasingly being explored for their potential to treat intestinal fibrosis (Figure 4). However, despite the promising direction, there is limited clinical evidence specifically addressing TCM’s efficacy in treating this condition. Ongoing clinical trials investigating TCM for intestinal fibrosis are in progress, with many drawing on the concept of syndrome differentiation (Zheng) that is central to TCM practice. This individualized approach could enhance therapeutic outcomes, as clinical evidence has demonstrated that addressing fibrosis according to TCM syndromes may improve disease progression. However, challenges remain, including the lack of robust evidence from large-scale, long-term studies, as well as the complexity and variability of TCM metabolites. Furthermore, the intricate mechanisms through which TCM works, and its interactions with modern pharmaceuticals, require further investigation. Moving forward, large-scale clinical trials with extended follow-up periods are essential to validate the efficacy of TCM in treating intestinal fibrosis. Additionally, identifying and isolating active metabolites from TCM will be crucial for advancing its therapeutic potential.

Changes in dietary habits are another significant factor influencing the development of IBD and subsequent fibrosis (Figure 4). A balanced diet, including supplementation with vitamins and trace elements, may play an important role in preventing the onset and progression of intestinal fibrosis. As we continue to explore anti-fibrotic targets and potential therapies, these findings highlight the need for integrated clinical strategies that combine pharmacological treatments with dietary and lifestyle interventions. Given that the underlying mechanisms of fibrosis share similarities across organs, insights from other fibrotic diseases can inform future research and help integrate TCM into mainstream clinical practice. By identifying key therapeutic targets and refining treatment strategies, these efforts hold promise for improving patient outcomes in the management of intestinal fibrosis.

## Glossary

AHR: Aryl Hydrocarbon Receptor
AIEC: adherent-invasive Escherichia coli
ASF: Altered Schaedler flora
α-SMA: α-smooth muscle actin
ATP: adenosine triphosphate
ATRA: All-trans retinoic acid
BHA: Betulinic acid hydroxamate
BM: bone marrow
CA: Calycosin
CARD-024: 1-alpha-hydroxyvitamin D5
CCA: Cinnamaldehyde
CCD-18Co cell: human normal colon epithelial cell
CCL11: chemokine (C-C motif) ligand 11
CCR3: C-C Chemokine Receptor Type 3
CD: Crohn's disease
CD4+: CD4+ T cells
CD8+: CD8+ T cells
CCE: Cinnamomum cassia extract
CTGF: Connective tissue growth factor
CT26: BALB/C mouse CT26 colon cancer cell
CUMS: chronic unpredictable mild stress;
Curcumin: [1,7-bis(4-hydroxy-3-methoxyphenyl)-1,6-heptadiene-3,5-dione]
CXCL8: KC/C-X-C motif ligand8
DAI: Disease activity index;
DIS: decellularized intestinal scaffolds
DKT: Da-jian-zhong-tang (Daikenchuto)
DNBS: TNBS is similar and more likely to produce MAT
DR3: Death Receptor 3
DSS: Dextran Sulfate Sodium Salt
eATP: extracellular adenosine triphosphate
EBD: endoscopic balloon dilation
ECM: extracellular matrix
MFGE8: milk fat globule-epidermal growth factor 8
EGR-1: early growth response gene -1
EMT: epithelial-mesenchymal transition
EMMPRIN: ECM metalloproteinase inducer
ES: endoscopic strictureplasty
ET-1: Endothelin-1
EVs: extracellular vesicles;
FAK: focal adhesion kinase
FICZ: Administration of 6-formylindolo(3,2-b) carbazole
FL-BsAb1/17: anti-colitis effects of bispecific antibody targeting IL-1β and IL-17A
Fn1: Fibronectin
FRB: Fermented Rice Bran
FS CD: fibrostenotic CD
FSP1: fibroblast-specific protein 1
GED: PPAR-γ modulator GED-0507-34 Levo
GF mice: Germ-Free Mice
GM-CSF: Granulocyte-Macrophage Colony-Stimulating Factor
hADSCs: human adipose-derived MSCs
HFD: high-fat diet
HIC-5: Hydrogen peroxide-inducible clone 5
HIF: Hypoxia-Inducible Factor
HIF-1α: HIF-1-alpha
HIFs: Human intestinal myofibroblasts
HIMFs: human primary intestinal myofibroblasts
HMC-1 cell: Human MCs
H_2_O_2_: hydrogen peroxide
HSP47: Heat shock protein
IBD: Inflammatory bowel disease
ICD: inflammatory CD
IECs: human intestinal epithelial cells
IEC-6 cell: Rat Small Intestine Crypt Epithelial Cells
IFN-γ: interferon-gamma
IGF-1: colonic insulin-like growth factor-I
ILCs: innate lymphoid cells;
IL1RL2 or IL36R: Interleukin-1 Receptor-Like 2
IL-1β: interleukin 1β
IL-17: interleukin 17
IMEF cell: immortalized mouse embryonic fibroblasts
InMyoFib cell: intestinal myofibroblast cell
iNOS: inducible nitric oxide synthase
IR: 13 Gy of IR using an X-RAD320 system
ISMCs: intestinal smooth muscle cells
Itch: Itchy E3 ubiquitin-protein ligase
Itch^-/-^ mice: Itch-deficient mice
ITE: 2-(10H-indole-30-carbonyl)-thiazole-4-carboxylic acid methyl ester
JNK: c-Jun N-terminal kinase
LAD-2 cells: Human MCs
L-Tg mice: Mice with elevated T-cell expression of Tl1A
MAT: Mesenteric adipose tissue
MAPK: Mitogen-Activated Protein Kinase
MCs: Mast cells
M-CSFR-1: IL-34 receptor
MCP-1: monocyte chemoattractant protein-1
MCT: mast cell tryptase
ME: maggot extract
MFE40: the 40% ethanol fraction of Mume Fructus
MLCK: myosin light chain kinase
MMP-9: matrix metalloprotein 9
MPFs: Mouse Primary Fibroblasts
MRTF-A: Myocardin-Related Transcription Factor-A
MSCs: mesenchymal cell
MSC-EVs: mesenchymal stromal cells secreted extracellular vesicles
MSC-Exos: human bone marrow-derived mesenchymal stromal cells
NIH-3T3-EPO-luc cell: NIH-3T3 fibroblasts stably transfected with a luciferase-HRE
NMN: Nicotinamide mononucleotide
NOX4: NADPH Oxidase 4
Nrf2: Nuclear factor erythroid2-related factor 2
PAR-2: Protease-Activated Receptor-2
PDGF: platelet-derived growth factor receptor-beta
PFD: Pirfenidone (5-methyl-N-phenyl-2-(1H)-pyridone)
p-MLC: phosphorylated Myosin Light Chain
PPAR-γ: peroxisome proliferator-activated receptor-γ
PTGS2: encode the COX-2 enzyme
RAG-/- mice: gene-1 deficient mice
RAS: renin-angiotensin system
RIF: Radiation-induced intestinal fibrosis
RIFs: Primary small intestinal fibroblasts from rats
RORγ-t: retinoic acid receptor-related orphan receptor gamma-t
SCFAs: short-chain fatty acids
SD Rat: Sprague Dawley Rat
SEMFs: subepithelial myofibroblast
SI: small intestine
Silybum marianum: The fruits and seeds of milk thistle
SM: submucosal layer
SPF: Specific Pathogen-Free
SRF: Serum Response Factor
SUCNR1: succinate receptor;
TCM: Traditional Chinese Medicine
TF: tissue factor
TFA: Total flavone of Abelmoschus manihot
TGF-β1: Transforming growth factor beta-1
TIMP-1: tissue inhibitor of metalloproteinases-1
TJ: tight junction
TL1A: Tumor Necrosis Factor-like Ligand 1A
TNBS: 2,4,6-Trinitrobenzenesulfonic acid solution
TNFSF15: Tumor Necrosis Factor Superfamily Member 15
TNF-α: tumor necrosis factor-α
Tpsb1: tryptase genes
Tpsb2: tryptase genes
TRG/RSG: troglitazone and rosiglitazone
TRPA1: transient receptor potential ankyrin1
UC: ulcerative colitis
UC/PL-MSCs: Umbilical cord /placenta-derived MSCs
VDR: Vitamin D Receptor
VEGF: vascular endothelial growth factor
ZEB1: Zinc Finger E-box Binding Homeobox 1
ZNFs: Zinc-finger proteins
8-OHG: 8-hydroxyguanosine
